# Mapping of *Mycobacterium tuberculosis* Complex Genetic Diversity Profiles in Tanzania and Other African Countries

**DOI:** 10.1371/journal.pone.0154571

**Published:** 2016-05-05

**Authors:** Erasto V. Mbugi, Bugwesa Z. Katale, Elizabeth M. Streicher, Julius D. Keyyu, Sharon L. Kendall, Hazel M. Dockrell, Anita L. Michel, Mark M. Rweyemamu, Robin M. Warren, Mecky I. Matee, Paul D. van Helden, David Couvin, Nalin Rastogi

**Affiliations:** 1 Department of Biochemistry, Muhimbili University of Health and Allied Sciences, P. O. Box 65001, Dar es Salaam, Tanzania; 2 Department of Microbiology and Immunology, Muhimbili University of Health and Allied Sciences, P.O. Box 65001, Dar es Salaam, Tanzania; 3 DST/NRF Centre of Excellence for Biomedical Tuberculosis Research/ South African Medical Research Council (MRC) Centre for Tuberculosis Research, Division of Molecular Biology and Human Genetics, Faculty of Health Sciences, Stellenbosch University, P.O. Box 241, Cape Town, 8000, South Africa; 4 Tanzania Wildlife Research Institute (TAWIRI), P.O. Box 661, Arusha, Tanzania; 5 Department of Immunology and Infection, London School of Hygiene and Tropical Medicine, Keppel Street, London, WC1E 7HT, United Kingdom; 6 The Royal Veterinary College, Royal College Street, London, NW1 0TU, United Kingdom; 7 Department of Veterinary Tropical Diseases, Faculty of Veterinary Science, University of Pretoria, Pretoria, South Africa; 8 Southern African Centre for Infectious Disease Surveillance, Sokoine University of Agriculture, Morogoro, Tanzania; 9 WHO Supranational TB Reference Laboratory, Tuberculosis & Mycobacteria Unit, Institut Pasteur de la Guadeloupe, Morne Joliviere, BP 484, 97183, Abymes, Guadeloupe; University of Minnesota, UNITED STATES

## Abstract

The aim of this study was to assess and characterize *Mycobacterium tuberculosis* complex (MTBC) genotypic diversity in Tanzania, as well as in neighbouring East and other several African countries. We used spoligotyping to identify a total of 293 *M*. *tuberculosis* clinical isolates (one isolate per patient) collected in the Bunda, Dar es Salaam, Ngorongoro and Serengeti areas in Tanzania. The results were compared with results in the SITVIT2 international database of the Pasteur Institute of Guadeloupe. Genotyping and phylogeographical analyses highlighted the predominance of the CAS, T, EAI, and LAM MTBC lineages in Tanzania. The three most frequent Spoligotype International Types (SITs) were: SIT21/CAS1-Kili (n = 76; 25.94%), SIT59/LAM11-ZWE (n = 22; 7.51%), and SIT126/EAI5 tentatively reclassified as EAI3-TZA (n = 18; 6.14%). Furthermore, three SITs were newly created in this study (SIT4056/EAI5 n = 2, SIT4057/T1 n = 1, and SIT4058/EAI5 n = 1). We noted that the East-African-Indian (EAI) lineage was more predominant in Bunda, the Manu lineage was more common among strains isolated in Ngorongoro, and the Central-Asian (CAS) lineage was more predominant in Dar es Salaam (p-value<0.0001). No statistically significant differences were noted when comparing HIV status of patients vs. major lineages (p-value = 0.103). However, when grouping lineages as Principal Genetic Groups (PGG), we noticed that PGG2/3 group (Haarlem, LAM, S, T, and X) was more associated with HIV-positive patients as compared to PGG1 group (Beijing, CAS, EAI, and Manu) (p-value = 0.03). This study provided mapping of MTBC genetic diversity in Tanzania (containing information on isolates from different cities) and neighbouring East African and other several African countries highlighting differences as regards to MTBC genotypic distribution between Tanzania and other African countries. This work also allowed underlining of spoligotyping patterns tentatively grouped within the newly designated EAI3-TZA lineage (remarkable by absence of spacers 2 and 3, and represented by SIT126) which seems to be specific to Tanzania. However, further genotyping information would be needed to confirm this specificity.

## Introduction

Human tuberculosis remains a global leading devastating, often severe and contagious chronic respiratory disease. People with immune-suppression are more susceptible to tuberculosis (TB). With the Global Tuberculosis Report indicating 13% of new TB cases being HIV-positive worldwide and up to 78% of TB cases among people living with HIV worldwide found in African region [1], the disease impact becomes further magnified especially in developing countries. In Tanzania various studies have been conducted using both conventional and non-conventional means to characterize the disease and its spread. Some of these studies focus on epidemiology [2–5], diagnostics [6–9], treatment and control [2, 10–12], strain molecular characterization [13–16], TB-HIV co-infections [17–20], challenges and resource limitation [21], and more recently, TB cross-species transmission at the human-animal interface [22–24]. In the recent study by our group in Serengeti ecosystem [15], a strain unassigned to neither of known spoligotypes, but resembling the CAS strain family was identified and tentatively named ‘Serengeti strain’. Although various TB studies have been conducted in Tanzania so far, none of them has given detailed information on all the major phylogenetic lineages of tubercle bacilli and their distribution. In addition, none of the studies compared Tanzanian strain patterns with those prevailing in neighbouring countries and sub-regions to underline differences relating to the presence of specific lineages. All this information is important in establishing phylogenetical relatedness of the strains causing disease within and between countries as well as establishing transmission links between individuals. This study aimed to specifically describe the genotypic diversity of *Mycobacterium tuberculosis* complex (MTBC) in Tanzania, as well as neighbouring and other countries in Africa (having spoligotyping data deposited in SITVIT database), with reference to their global distribution. The study also assessed the association between HIV serological status and *M*. *tuberculosis* lineages for people who apart from TB had HIV. To better highlight region-specificity or similarity and get a larger view on MTBC distribution in Tanzania, *Mycobacterium tuberculosis* complex (MTBC) genotypic diversity was characterized and compared to diverse genotypes available in other African countries. Basing on genotyping information, this paper also tentatively proposes a sublineage which seems to be phylogeographically specific for Tanzania.

## Materials and Methods

### Study areas

The Tanzanian studies were conducted in two geographically different areas. First is the Serengeti ecosystem comprised of Bunda, Serengeti and Ngorongoro districts in northern Tanzania [15]. The second is the Dar es Salaam region, along the coast in the eastern zone (newly reported). Dar es Salaam is located at 6°48' South, 39°17' East (−6.8000, 39.2833), along the natural harbour on the eastern coast of Africa, with sandy beaches in some areas (Population Distribution by Administrative Units, United Republic of Tanzania, 2013). Population density in Dar es Salaam is 3,133 persons per square kilometer. The increased birth and immigration rates as well as transient populations largely influence the relatively high population rate. The population densities and annual growth rates (in brackets) for Bunda, Serengeti and Ngorongoro in persons per square kilometers, are 70 (1.8%), 22.4 (3.5%) and 11.2 (3.0%), respectively.

### Neighbouring countries

The main neighboring countries of Tanzania are Kenya and Uganda in the north; Rwanda, Burundi, and the Democratic Republic of the Congo in the west; and Zambia, Malawi, and Mozambique in the south (http://en.wikipedia.org/wiki/Tanzania). The description of *M*. *tuberculosis* strain profile used the findings from the current study in Tanzania by Mbugi et al [15] and already published data of existing patterns from other countries in the international genotyping database SITVIT2.

### Preliminary Laboratory sample processing and Ethics Approval

All preliminary samples processing post field collection for Tanzanian study was performed at the Infectious Disease Centre (IDC) and Muhimbili University of Health and Allied Sciences (MUHAS) in Dar es Salaam. Spoligotyping was done at the DST/NRF Centre of Excellence for Biomedical Tuberculosis Research/South African Medical Research Council (MRC) Centre for Tuberculosis Research, Division of Molecular Biology and Human Genetics, Faculty of Health Sciences, Stellenbosch University, South Africa. Details, as regards the whole study (including location and study design) for the Serengeti ecosystem, and lab analysis for all samples, have been previously reported [15]. Ethical approval for this study was obtained from the Muhimbili University of Health and Allied Sciences (MUHAS) Ethics Review Committee (Ref.MU/PGS/PhD/R/Vol.1) and The Tanzania National Institute for Medical Research (Ref. No. NIMR/HQ/R.8a/Vol. IX/1299). Participants consented to enrol in the study after completing informed consent forms. When patients were found to have tuberculosis, treatment was offered as per the Tanzanian National Guidelines for management of tuberculosis. Information on other infections than tuberculosis was also collected from patients and of particular interest, was HIV serology.

### Spoligotypes and new names assignment

Spoligotyping was performed using a commercially available spoligotyping kit (Isogen, Bioscience BV, Maarssen, The Netherlands) as previously described by Kamerbeek et al [25]. The resulting spoligotypes were reported in octal and binary formats (**Table 1**) and compared to existing patterns in an international genotyping database SITVIT2, which is an updated version of previously released SITVITWEB database [26] available at: http://www.pasteur-guadeloupe.fr:8081/SITVIT_ONLINE/. Spoligotype patterns were grouped as spoligotype international types (SITs) if they shared identical spoligotype patterns with patterns present in the existing database. Spoligotypes that had no match with previously reported patterns, represented by unique exemplars, were considered as orphans. The database provided tools for queries and conversion into binary or octal values of spoligotypes, as well as geographical distribution maps for worldwide comparisons. Spoligotyping data obtained from Tanzania were compared with patterns from neighbouring countries and sub-regions in order to underline differences relating to the presence or absence of specific lineages. This includes the ‘Serengeti strains’ that were recently reported in Serengeti ecosystem [15].

**Table 1:** Distribution of lineages/sublineages of isolates from Tanzania (n = 293).

Lineage/Sublineage	Number	%
**Beijing**	**12**	**4,10**
**CAS**	**105**	**35,84**
CAS1-Delhi	19	6,48
CAS1-Kili	78	26,62
CAS2	2	0,68
CAS (or CAS_Like)	6	2,05
**EAI**	**49**	**16**,**72**
EAI1-SOM	3	1,02
EAI5	39	13,31
EAI6-BGD1	2	0,68
EAI8-MDG	2	0,68
EAI (or EAI_Like)	3	1,02
**Haarlem**	**7**	**2,39**
H1	2	0,68
H3	5	1,71
**LAM**	**43**	**14,68**
LAM6	2	0,68
LAM9	9	3,07
LAM11-ZWE	32	10,92
**Manu**	**10**	**3,41**
Manu_ancestor	1	0,34
Manu1	2	0,68
Manu2	7	2,39
**S**	**1**	**0,34**
**T**	**64**	**21,84**
T1	29	9,90
T2-uganda	19	6,48
T3	4	1,37
T3-ETH	11	3,75
T (or T_Like)	1	0,34
**X**	**1**	**0,34**
X2	1	0,34
**Unknown**	**1**	**0,34**

### Data analysis

Data were entered using Microsoft Excel and later transferred to relevant software for analysis. STATA version 12 and MegaStat softwares were used for this statistical analysis and a p-value <0.05 was considered significant. Standard deviation (SD) and means were calculated for age of patients. Odds ratio (OR) and 95% confidence interval (CI) were calculated for comparison between HIV information versus PGG1 and PGG2/3 lineage groups. The spoligotype patterns from Dar es Salaam and the Serengeti ecosystem study were pooled together and reanalysed for comparison with already known patterns from neighbouring countries (Kenya, Uganda, Zambia, Malawi, and Mozambique), other African countries (Sudan, Ethiopia, Nigeria, Cameroon, Namibia, South Africa, Zimbabwe, and Madagascar) and those available globally in SITVIT2 database.

### Phylogenetic analysis and mapping

MLVA Compare V1.03 software (Genoscreen; Lille, France) was used to draw the minimum spanning trees (MSTs). The SIT or orphan spoligotype number appeared inside each node, and the distance (number of spacers of difference) between two nodes was shown on the edge linking these nodes. These phylogenetic trees were coloured in function of various characteristics such as the MTBC lineages described in SITVIT, the cities of isolation, and the HIV serological data of patients. The MST is a graph which is undirected and connected. The MST links all isolates together with the fewest possible linkages between nearest neighbours. Furthermore, a spoligoforest was drawn as a “hierarchical layout” using the SpolTools software (available through http://www.emi.unsw.edu.au/spolTools; [27, 28]). As opposed to the MST, the spoligoforest is a directed and not necessarily connected phylogenetic tree illustrating the parent to descendant relationships between spoligotypes (considering the fact that spoligotypes rather evolve by loss of spacers). TBVis tool (available at http://tbinsight.cs.rpi.edu/; [29, 30]) was used to visualize and map the spoligotypes shared between different lineages and split by city of isolation and the information of HIV serology of patients. Maps were reproduced and designed according to terms described in the Creative Commons 3.0 Attribution License (http://creativecommons.org/licenses/by/3.0/).

## Results

### Distribution of cities of isolation and lineages in Tanzanian study

From a total of 293 Tanzanian isolates, 93 (31.74%) were collected from Ngorongoro; 79 (26.96%) were collected from Dar es Salaam; 77 (26.28%) were collected from Bunda; and 44 (15.02%) were collected from Serengeti. Strains belonging to CAS lineage were found in highest proportion in this study (n = 105 or 35.8% of isolates), followed by ill-defined T lineage strains (n = 64 or 21.8% of isolates). The proportion of EAI lineage strains (n = 49 or 16.72% of isolates) was not negligible (**Table 1**). Among the EAI lineage, EAI5 sublineage was found in relatively higher proportion (n = 39 or 13.31% of all isolates). However, our findings suggest that a high proportion of strains belonging to the newly defined EAI3-TZA sublineage (which seems to be specific to Tanzania) were misclassified as EAI5 sublineage by SITVIT2.

### HIV Status, age, and Sex ratio

HIV serology was known for 214 patients: HIV-positive patients accounted for 21 cases, and HIV-negative accounted for 193 cases. No significant statistical differences were noted when comparing lineage distribution vs. HIV serological status (p-value = 0.103), however a significant proportion of serologically HIV-positive patients was associated with the LAM and T lineage strains (n = 6/38 or 15.79% and 7/57 or 12.28%, respectively) based on known HIV status for each lineage. Furthermore, we noticed that PGG2/3 group also defined as the Euro-American family (Haarlem, LAM, S, T, and X) was more associated with HIV-seropositive patients as compared to PGG1 group (Beijing, CAS, EAI, and Manu). Among PGG2/3, 15 patients were HIV-seropositive vs. 89 HIV-seronegative; as compared to 6 HIV-seropositive patients vs. 103 HIV-seronegative patients among PGG1 group (p-value = 0.03; OR = 2.88, 95%CI [1.003–9.4544]).

The mean age of the patients was 35.1 years old, and median age was 35 years old (**Table 2**). The male/female sex ratio was 156/137 or 1.14. HIV serology distribution was not significantly different between female and male patients (p-value = 0.931); neither between the various cities of isolation (p-value = 0.921).

**Table 2:** Descriptive statistics on age of patients in this study.

* Descriptive statistics on*	*Age*
Count	293
Mean	35,11
Sample variance	101,56
Sample standard deviation	10,08
Minimum	18
Maximum	75
Range	57
1st quartile	27,00
Median	35,00
3rd quartile	41,00
Interquartile range	14,00
Mode	39,00

No significant statistical differences were noted when comparing lineage distribution vs. gender/sex of the patients (p-value = 0.653).

### Distribution of Spoligotype International Types (SITs) in this study

A total of 54/57 SITs containing 264 isolates matched a pre-existing shared-type in the SITVIT2 database, whereas 3/57 SITs (n = 4 isolates) were newly created. A total of 27/57 SITs containing 238 isolates were clustered within this study (2 to 76 isolates per cluster) while 30/57 SITs containing 30 isolates were unique (in addition 25 orphan strains were identified, which brings the number of unclustered isolates in this study to 55/293 or 18.8%, and clustered isolates to 238/293 or 81.2%). The distribution of Spoligotype International Types (SITs) in this study is shown in **Table 3**.

**Table 3:** Distribution of Spoligotype International Types (SIT) in this study.

SIT*	Spoligotype pattern (Octal format)	Number in study (%)	% in study vs. Database	Lineage**	Unique or Clustered SIT***
1	000000000003771	12 (4.1)	0.11	Beijing	Clustered
4	000000007760771	1 (0.34)	0.26	Unknown	Unique
8	400037777413771	12 (4.1)	6	EAI5	Clustered
10	477777277413771	1 (0.34)	1.12	EAI8-MDG	Unique
21	703377400001771	76 (25.94)	15.97	CAS1-Kili	Clustered
22	703777400001771	1 (0.34)	1.08	CAS1-Delhi	Unique
25	703777740003171	3 (1.02)	0.45	CAS1-Delhi	Clustered
26	703777740003771	14 (4.78)	0.8	CAS1-Delhi	Clustered
34	776377777760771	1 (0.34)	0.11	S	Unique
36	777737777720771	1 (0.34)	0.63	H3	Unique
37	777737777760771	4 (1.37)	0.73	T3	Clustered
42	777777607760771	6 (2.05)	0.17	LAM9	Clustered
48	777777777413731	1 (0.34)	0.22	EAI1-SOM	Unique
50	777777777720771	3 (1.02)	0.07	H3	Clustered
53	777777777760771	15 (5.12)	0.23	T1	Clustered
59	777777606060771	22 (7.51)	4.54	LAM11-ZWE	Clustered
62	777777774020731	1 (0.34)	0.17	H1	Unique
64	777777607560771	2 (0.68)	0.48	LAM6	Clustered
73	777737777760731	1 (0.34)	0.34	T	Unique
126	477777777413771	18 (6.14)	12.08	EAI5	Clustered
128	637775777760730	3 (1.02)	8.57	T2-uganda	Clustered
135	777777777760730	4 (1.37)	11.43	T2-uganda	Clustered
137	777776777760601	1 (0.34)	0.1	X2	Unique
149	777000377760771	4 (1.37)	0.82	T3-ETH	Clustered
244	777777777760601	5 (1.71)	4.03	T1	Clustered
245	777777777760671	1 (0.34)	4.55	T1	Unique
281	777775777760771	1 (0.34)	3.13	T1	Unique
288	700377740003771	2 (0.68)	1.27	CAS2	Clustered
345	777000377760731	7 (2.39)	33.33	T3-ETH	Clustered
420	637774777760730	6 (2.05)	24	T2-uganda	Clustered
458	777777777403771	1 (0.34)	2.7	EAI5	Unique
486	703777740000371	1 (0.34)	1.75	CAS	Unique
522	777777777760770	1 (0.34)	6.25	T1	Unique
523	777777777777771	1 (0.34)	1.69	Manu_ancestor	Unique
702	700775747413771	1 (0.34)	2.86	EAI6-BGD1	Unique
727	777737774020731	1 (0.34)	2.44	H1	Unique
815	777777606060731	2 (0.68)	1.34	LAM11-ZWE	Clustered
891	777777607660771	2 (0.68)	6.67	LAM9	Clustered
1090	077777777413771	2 (0.68)	22.22	EAI5	Clustered
1192	777777677763771	1 (0.34)	10	Manu2	Unique
1369	477777777413671	1 (0.34)	12.5	EAI5	Unique
1549	775777606060731	2 (0.68)	18.18	LAM11-ZWE	Clustered
1765	703337400001771	1 (0.34)	14.29	CAS1-Kili	Unique
1801	777777777413730	1 (0.34)	25	EAI1-SOM	Unique
1824	760377777760701	2 (0.68)	25	T1	Clustered
1957	477777777013771	1 (0.34)	6.67	EAI5	Unique
2196	777775606060731	6 (2.05)	30	LAM11-ZWE	Clustered
2267	701377400001771	1 (0.34)	25	CAS	Unique
2269	703377400000771	1 (0.34)	20	CAS	Unique
2364	703601740003771	1 (0.34)	25	CAS1-Delhi	Unique
2391	703377400003771	1 (0.34)	11.11	CAS1-Kili	Unique
2484	737377677760771	1 (0.34)	25	T1	Unique
2736	777777777760741	1 (0.34)	25	T1	Unique
3880	437774777760730	2 (0.68)	50	T2-uganda	Clustered
4056*	477777777413411	2 (0.68)	100	EAI5	Clustered
4057*	777017777760771	1 (0.34)	50	T1	Unique
4058*	477777777413371	1 (0.34)	50	EAI5	Unique

* A total of 54/57 SITs containing 264 isolates matched a preexisting shared-type in the database, whereas 3/57 SITs (n = 4 isolates) were newly created. A total of 27/57 SITs containing 238 isolates were clustered within this study (2 to 76 isolates per cluster) while 30/57 SITs containing 30 isolates were unique (for total unique strains, one should add to this number the 25 orphan strains, which brings the number of unclustered isolates in this study to 55/293 or 18.77%, and clustered isolates to 238/293 or 81.23%). Note that SITs followed by an asterisk indicates "newly created” SITs due to 2 or more strains belonging to an identical new pattern within this study or after a match with an orphan in the database; SIT designations followed by number of strains: 4056* this study n = 2; 4057* this study n = 1, SDN n = 1; 4058* this study n = 1, NLD n = 1.

** Lineage designations according to SITVIT2; “Unknown” designates patterns with signatures that do not belong to any of the major lineages described in the database.

*** Clustered strains correspond to a similar spoligotype pattern shared by 2 or more strains “within this study”; as opposed to unique strains harboring a spoligotype pattern that does not match with another strain from this study. Unique strains matching a preexisting pattern in the SITVIT2 database are classified as SITs, whereas in case of no match, they are designated as “orphan”.

### Clusters containing 3 or more isolates and their worldwide distribution in the SITVIT2 database

The results from Dar es Salaam and the Serengeti ecosystem studies were collectively analyzed and proportions of various lineages estimated to represent TB lineages potentially found in Tanzania. There were no major differences from what was reported in a separate Serengeti ecosystem and the combined results, which included samples from Dar es Salaam study [14]. A very high proportion of SIT21/CAS1-Kili was recorded in this pooled study (n = 76 or 25.9% of isolates) (**Table 3**). The description of SIT/lineages and worldwide distribution, as compared to the SITVIT2 database, of clusters containing 3 or more isolates is presented in Table 4. Apart from SIT21/CAS1-Kili, other SITs/lineages were observed in the order from high to low proportion as SIT59/LAM11-ZWE, SIT126/EAI5, SIT53/T1, SIT26/CAS1-Delhi, SIT1/Beijing, SIT8/EAI5, SIT345/T3-ETH, SIT42/LAM9, SIT420/T2-Uganda, SIT2196/LAM11-ZWE, SIT244/T1, SIT37/T3, SIT135/T2-Uganda, SIT149/T3-ETH, SIT25/CAS1-Delhi, SIT50/H3 and SIT128/T2-Uganda.

**Table 4:** Description of clusters containing 3 or more isolates in this study, and their worldwide distribution in the SITVIT2 database.

SIT/Lineage	Number in study	% in study	% in study vs. database	Distribution in Regions with > = 3% of a given SITs *	Distribution in countries with > = 3% of a given SITs **
21/CAS1-Kili	76	25.94	15.97	AFRI-E 65.97, EURO-N 10.08, EURO-W 6.3, ASIA-W 5.25, AMER-N 5.25, AFRI-S 4.83	TZA 34.66, ETH 9.87, MDG 9.66, USA 5.25, ZAF 4.83, ZMB 4.62, SWE 3.99, KEN 3.57
59/LAM11-ZWE	22	7.51	4.54	AFRI-E 58.35, AFRI-S 15.26, EURO-W 14.64, AMER-N 3.3	ZMB 21.86, ZWE 15.88, ZAF 15.26, BEL 13.4, TZA 11.13, USA 3.3, MWI 3.3
126/EAI5	18	6.14	12.08	ASIA-S 37.58, ASIA-W 16.78, AFRI-E 15.44, EURO-N 13.42, EURO-W 5.37, ASIA-SE 4.03, AMER-N 4.03	IND 30.2, TZA 14.77, OMN 11.41, GBR 10.07, SAU 5.37, NLD 4.7, LKA 4.7, USA 4.03
53/T1	15	5.12	0.23	AMER-S 14.97, EURO-W 14.82, AMER-N 12.77, EURO-S 8.91, EURO-N 7.09, ASIA-W 6.92, AFRI-S 4.7, AFRI-E 4.63, ASIA-E 4.04, AFRI-N 3.33, EURO-E 3.09, CARI 3.06, AMER-C 3.06	USA 12.5, FXX 7.46, BRA 5.55, ITA 5.05, ZAF 4.6, PER 3.69, TUR 3.29, AUT 3.24
26/CAS1-Delhi	14	4.78	0.8	ASIA-S 55.97, AMER-N 12.46, ASIA-W 12.12, AFRI-E 5.06, EURO-W 4.84, EURO-N 3.75	IND 38.91, USA 12.46, PAK 8.19, OMN 6.2, BGD 4.72, SAU 4.44, IRN 3.53
1/Beijing	12	4.1	0.11	ASIA-E 32.35, AMER-N 19.02, ASIA-SE 10.11, AFRI-S 7.85, ASIA-N 6.59, ASIA-S 5.64, EURO-N 3.46, AMER-S 3.44	CHN 19.22, USA 18.78, JPN 10.91, ZAF 7.85, RUS 6.59, VNM 3.69, IND 3.26, PER 3.0
8/EAI5	12	4.1	6	AFRI-E 21.5, ASIA-W 18.5, AMER-C 17.0, EURO-N 15.0, AMER-N 14.5, ASIA-S 4.0, AFRI-S 3.0	MEX 17.0, USA 14.5, OMN 14.5, TZA 11.5, DNK 8.0, MOZ 6.0, GBR 5.0, ZMB 4.0, IND 4.0, ZAF 3.0, SAU 3.0
345/T3-ETH	7	2.39	33.33	AFRI-E 57.14, EURO-N 33.33, AMER-N 9.52	TZA 33.33, GBR 19.05, ETH 14.29, USA 9.52, KEN 9.52, FIN 9.52, NOR 4.76
42/LAM9	6	2.05	0.17	AMER-S 31.94, AMER-N 11.17, EURO-S 10.66, EURO-W 8.92, AFRI-N 8.07, EURO-N 4.62, CARI 4.0, AFRI-E 3.52, AMER-C 3.35	BRA 12.89, USA 11.17, COL 7.18, MAR 6.64, ITA 6.16, FXX 4.76, PER 3.49, ESP 3.15, VEN 3.12
420/T2-uganda	6	2.05	24	AFRI-E 40.0, AMER-N 32.0, EURO-W 24.0, EURO-N 4.0	USA 32.0, TZA 32.0, BEL 20.0, ZMB 4.0, UGA 4.0, SWE 4.0, DEU 4.0
2196/LAM11-ZWE	6	2.05	30	AFRI-E 70.0, AFRI-S 20.0, EURO-W 5.0, EURO-N 5.0	TZA 35.0, MOZ 25.0, ZAF 20.0, ZMB 10.0, FIN 5.0, BEL 5.0
244/T1	5	1.71	4.03	AFRI-E 16.13, AFRI-S 15.32, EURO-S 14.52, AMER-S 14.52, ASIA-S 12.9, EURO-W 12.1, AFRI-W 6.45, AMER-N 4.03	ZAF 15.32, PRT 12.9, BRA 12.9, BGD 11.29, FXX 10.48, ZMB 6.45, GNB 5.65, TZA 4.84, USA 4.03
37/T3	4	1.37	0.73	AFRI-E 18.98, AMER-S 12.96, EURO-N 11.31, EURO-W 10.95, ASIA-W 10.04, AMER-N 8.21, ASIA-E 5.84, EURO-S 5.47, EURO-E 4.2, ASIA-S 3.47, AFRI-S 3.1	ETH 15.88, USA 7.3, CHL 5.47, SWE 4.93, SAU 4.75, CHN 4.75, FXX 4.01, ITA 3.83, BRA 3.47, ZAF 3.1, DNK 3.1
135/T2-uganda	4	1.37	11.43	EURO-W 34.29, AMER-N 17.14, AFRI-S 17.14, AFRI-E 17.14, ASIA-W 5.71	ZAF 17.14, USA 17.14, TZA 11.43, DEU 11.43, NLD 8.57, AUT 8.57, SAU 5.71, FXX 5.71
149/T3-ETH	4	1.37	0.82	AFRI-E 58.4, EURO-N 21.52, ASIA-W 6.97, EURO-W 6.35, AMER-N 4.71	ETH 57.58, DNK 11.68, SAU 6.56, USA 4.71, SWE 4.51, NLD 3.48
25/CAS1-Delhi	3	1.02	0.45	ASIA-W 25.26, AFRI-E 23.76, ASIA-S 17.59, AFRI-N 12.63, AMER-N 7.82, EURO-N 5.41, EURO-W 4.06	ETH 22.26, SAU 14.14, IND 13.38, SDN 10.38, USA 7.82, OMN 6.32, IRQ 4.36
50/H3	3	1.02	0.07	AMER-S 26.56, EURO-W 14.86, AMER-N 14.86, EURO-S 9.77, CARI 4.92, EURO-E 4.67, EURO-N 4.62, AFRI-N 3.6, AFRI-S 3.42, AFRI-M 3.2	USA 14.84, PER 13.54, BRA 7.1, FXX 5.82, AUT 5.15, ITA 4.6, ESP 4.6, ZAF 3.42, CMR 3.15, CZE 3.1
128/T2-uganda	3	1.02	8.57	EURO-W 40.0, AFRI-S 25.71, AFRI-E 25.71	ZAF 25.71, TZA 17.14, FXX 14.29, DEU 11.43, ZMB 8.57, NLD 8.57, BEL 5.71

* Worldwide distribution is reported for regions with more than 3% of a given SITs as compared to their total number in the SITVIT2 database. The definition of macro-geographical regions and sub-regions (http://unstats.un.org/unsd/methods/m49/m49regin.htm) is according to the United Nations; Regions: AFRI (Africa), AMER (Americas), ASIA (Asia), EURO (Europe), and OCE (Oceania), subdivided in: E (Eastern), M (Middle), C (Central), N (Northern), S (Southern), SE (South-Eastern), and W (Western). Furthermore, CARIB (Caribbean) belongs to Americas, while Oceania is subdivided in 4 sub-regions, AUST (Australasia), MEL (Melanesia), MIC (Micronesia), and POLY (Polynesia). Note that in our classification scheme, Russia has been attributed a new sub-region by itself (Northern Asia) instead of including it among rest of the Eastern Europe. It reflects its geographical localization as well as due to the similarity of specific TB genotypes circulating in Russia (a majority of Beijing genotypes) with those prevalent in Central, Eastern and South-Eastern Asia.

** The 3 letter country codes are according to http://en.wikipedia.org/wiki/ISO_3166-1_alpha-3; countrywide distribution is only shown for SITs with ≥3% of a given SITs as compared to their total number in the SITVIT2 database.

### Phylogenetic lineages vs. demographic characteristics

The distribution of phylogenetic lineages by gender showed no significant difference (**Table 5**). Strains belonging to CAS lineage were more visible with highest proportion in Dar es Salaam followed by Ngorongoro with Bunda and Serengeti having nearly equal proportions. The proportion of strains belonging to the ill-defined T lineage was second highest, Bunda having the highest proportion, followed by Serengeti, Ngorongoro and Dar es Salaam. The EAI lineage was another lineage found in relatively high proportion predominantly in Bunda, Dar es Salaam and Ngorongoro, Serengeti having the least proportion. The LAM lineage strains were found in high proportions particularly in Ngorongoro and Bunda. For patients who had HIV in association with TB, the predominant strains seemed to be belonging to LAM and T lineages. Other strains were found in small proportions within specific demographic groups (**Table 5**).

**Table 5:** Distribution of phylogenetical lineages vs. demographic characteristics of patients and cities of isolation.

Lineage	Female	Male	Mean age [Min—Max]	SD	HIV–	HIV+	Bunda	Dar es Salaam	Ngoron-goro	Serengeti
Beijing	3	9	34.42 [19–51]	9.64	7	1	3	4	1	4
CAS	49	56	36.62 [21–75]	11.15	54	3	11	48	34	12
EAI	25	24	31.27 [19–54]	8.85	32	2	20	15	10	4
Haarlem	3	4	38.14 [30–45]	4.78	6	1	1	0	4	2
LAM	22	21	34.07 [18–53]	8.63	32	6	14	5	18	6
Manu	4	6	34.00 [22–53]	9.49	10	0	1	0	9	0
S	1	0	41.00 [41–41]	_	0	1	1	0	0	0
T	28	36	36.28 [20–63]	10.20	50	7	26	7	15	16
X	1	0	37.00 [37–37]	_	1	0	0	0	1	0
Unknown	1	0	25.00 [25–25]	_	1	0	0	0	1	0

SD = Standard deviation

### Distribution map of *Mycobacterium tuberculosis* lineages in African countries

**Fig 1:**
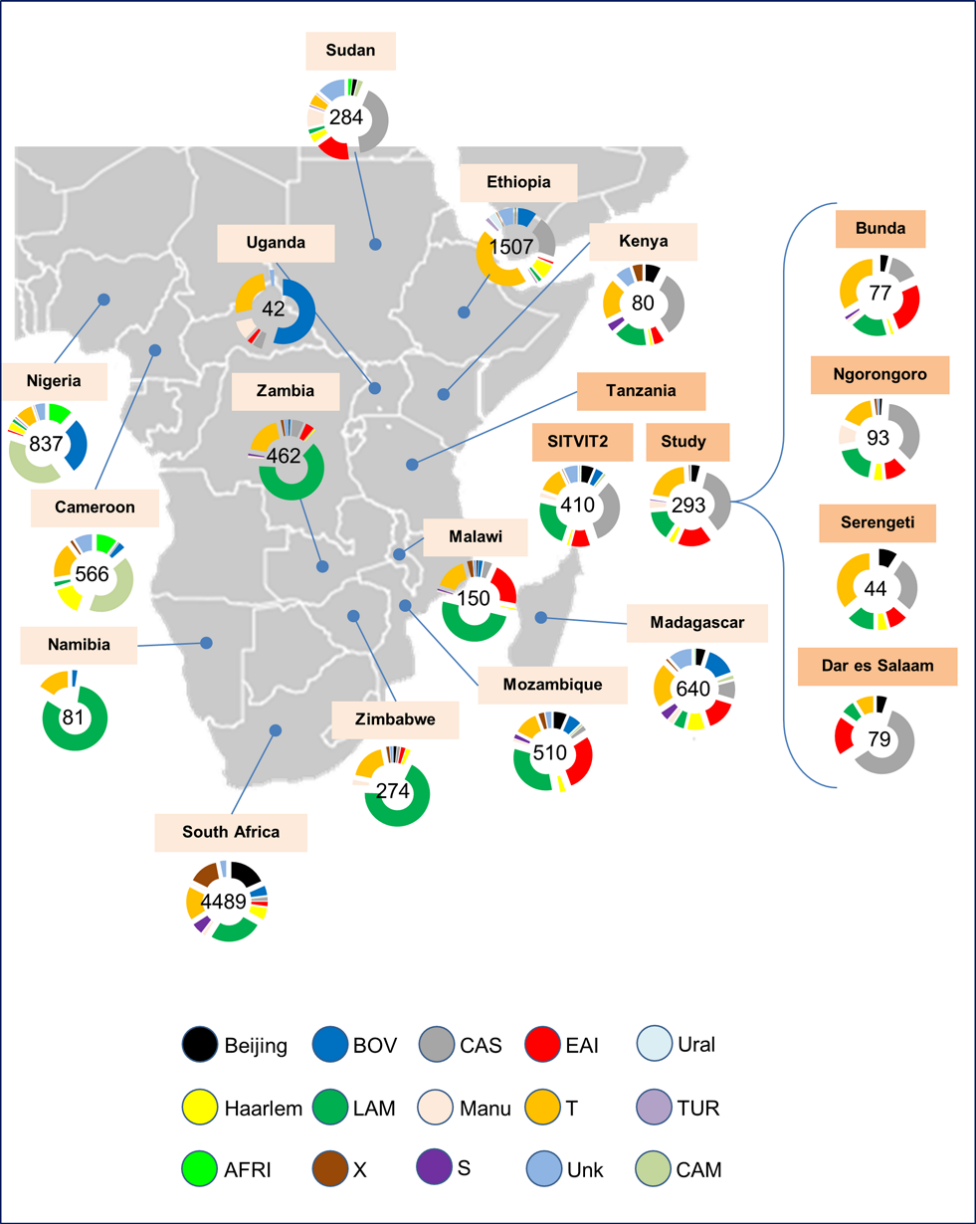
Distribution map of *Mycobacterium tuberculosis* lineages in several countries of Africa according to the SITVIT2 database.

Comparison of phylogenetic lineages between our study and those isolated in neighboring countries and other countries in Africa (representing a total of 9922 isolates) revealed the predominance of the CAS family especially in the north (Sudan) and Eastern region covering Ethiopia, Kenya, Tanzania and the Madagascar (Fig 1). Small proportions of CAS lineage were also recovered in Uganda, Zambia, Malawi and Mozambique. The EAI lineage was found to be more predominant in Mozambique, Malawi, Sudan, Tanzania and Madagascar with traces of this strain lineage found in Kenya, Uganda, Zambia, Zimbabwe and Ethiopia. The predominance of LAM lineage was notable in Namibia, Zimbabwe, Zambia, Malawi, Mozambique, South Africa, Kenya, Tanzania and Madagascar. Traces of this lineage were found sporadically in Sudan, Ethiopia, Cameroon, Nigeria and Uganda. The T family spread was notable covering all areas where data on TB in Africa were available; however, the smallest proportion of this lineage was noted in Sudan (Fig 1). The Beijing strain spreads out in the Eastern part of Africa (particularly Kenya, Tanzania, and Mozambique) all the way to South Africa including Madagascar Islands. The Haarlem family which was also detected in our study prevailed in Sudan, Ethiopia, Kenya, Madagascar, Mozambique, Zimbabwe, South Africa and Cameroon. The Manu family seemed to be more prevalent in Sudan and Uganda. The X family was also detected in studies in Tanzania with limited distribution though but it was better visible in South Africa and Kenya, and sporadically present in Mozambique, Malawi, Zambia, Zimbabwe, and Cameroon. Limited distribution of the S family was mostly found in South Africa, Madagascar, Mozambique and Kenya. Unknown lineage TB strains were found in most of the studied countries of Africa. The other lineages, CAM, TUR, AFRI and BOV which were reported in other African countries, were not detected in our Tanzanian study.

### Evolutionary relationships between M. tuberculosis spoligotypes

**Fig 2:**
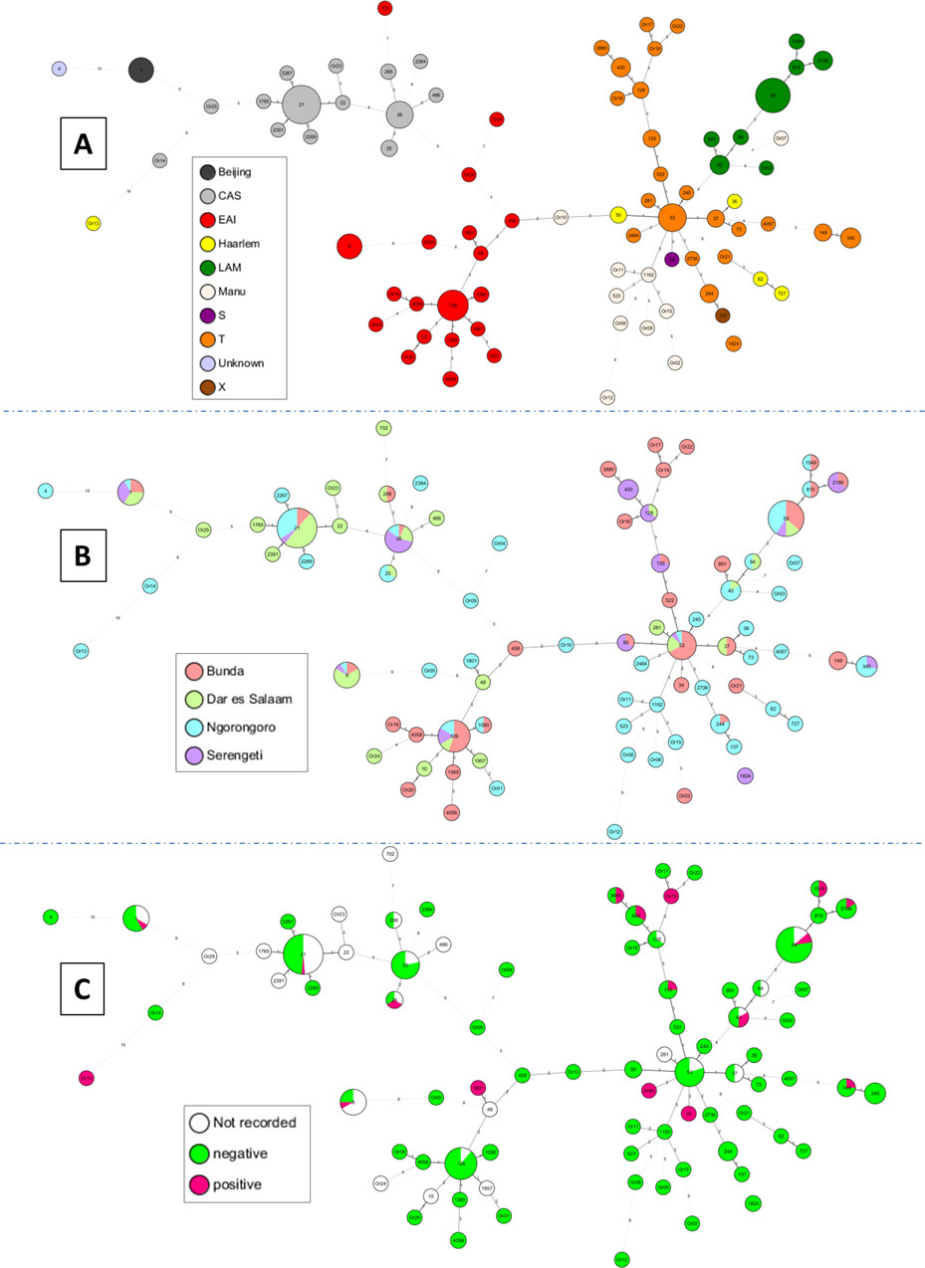
A minimum spanning tree (MST) illustrating evolutionary relationships between *M*. *tuberculosis* spoligotypes. MST constructed on all isolates including the orphan patterns (n = 293) in function of various characteristics. (**A**)MST based on phylogenetical lineages; (**B**) MST based on cities of isolation; and (**C**) MST based on HIV serology. The phylogenetic tree connects each genotype based on degree of changes required to go from one allele to another. The structure of the tree is represented by branches (continuous vs. dashed and dotted lines) and circles representing each individual pattern. Note that the length of the branches represents the distance between patterns while the complexity of the lines (continuous, gray dashed and gray dotted) denotes the number of allele/spacer changes between two patterns: solid lines, 1 or 2 or more changes (thicker ones indicate a single change, while the thinner one indicate 2 changes); gray dashed lines represent 3 changes; and gray dotted lines represent 4 or more changes. The size of the circle is proportional to the total number of isolates in our study, illustrating unique isolates (smaller nodes) versus clustered isolates (bigger nodes). The color of the circles indicates the phylogenetic lineage to which the specific pattern belongs.

Minimum spanning trees (MSTs) shown in Fig 2 were based on all spoligotypes of this study (n = 293 isolates). Fig 2A illustrated evolutionary relationships of spoligotypes in function of main MTBC lineages. The most visible lineages were CAS, T, EAI, and LAM. Among CAS lineage, the most predominant SITs were SIT21/CAS1-Kili (which is particularly endemic in Tanzania) and SIT26/CAS1-Delhi. Among EAI lineage, we can note the high prevalence of SIT126/EAI5 (tentatively relabeled EAI3-TZA) and SIT8/EAI5. SIT53/T1 was the better representative of the T lineage, and SIT59/LAM11-ZWE was predominant among LAM lineage. One may notice that the phylogenetic lineages were rather well organized in the MST (Fig 2A). Furthermore, the spoligoforest provided in S1 Fig allows a better visualization of main SITs and their tentative parent descendant relationships. As is for Fig 2A, Fig 2B also illustrated relationships among spoligotypes, but in function of the cities of isolation. This figure (Fig 2B) showed the distribution of spoligotypes as compared to the various cities of isolation present in this study (Bunda, Dar es Salaam, Ngorongoro, and Serengeti). Briefly, SIT21/CAS1-Kili was more common in Dar es Salaam followed by Ngorongoro; SIT26/CAS1-Delhi in Serengeti, SIT53/T1 in Bunda, SIT8/EAI5 in Dar es Salaam, SIT126/EAI3-TZA in Bunda, and SIT59/LAM11-ZWE both in Ngorongoro and Bunda (Fig 2B). Fig 2C illustrated phylogenetic relationships of spoligotypes in function of information on HIV serology of patients. We show that among the 82 distinct spoligotype patterns represented in this study, 17 patterns contained strains isolated from HIV-positive patients: i.e. SIT1/Beijing, SIT8/EAI5, SIT21/CAS1-Kili, SIT25/CAS1-Delhi, SIT34/S, SIT42/LAM9, SIT59/LAM11-ZWE, SIT135/T2-Uganda, SIT149/T3-ETH, SIT420/T2-Uganda, SIT1549/LAM11-ZWE, SIT1801/EAI1-SOM, SIT2196/LAM11-ZWE, SIT2484/T1, SIT3880/T2-uganda, including two orphan patterns Or13 and Or19. A TBVis graph representation is provided in S1 Fig, to illustrate the distribution of spoligotyping patterns and lineages as compared to city of isolation and HIV serological information of patients.

### Spoligotype geographic distribution and migration routes

**Fig 3:**
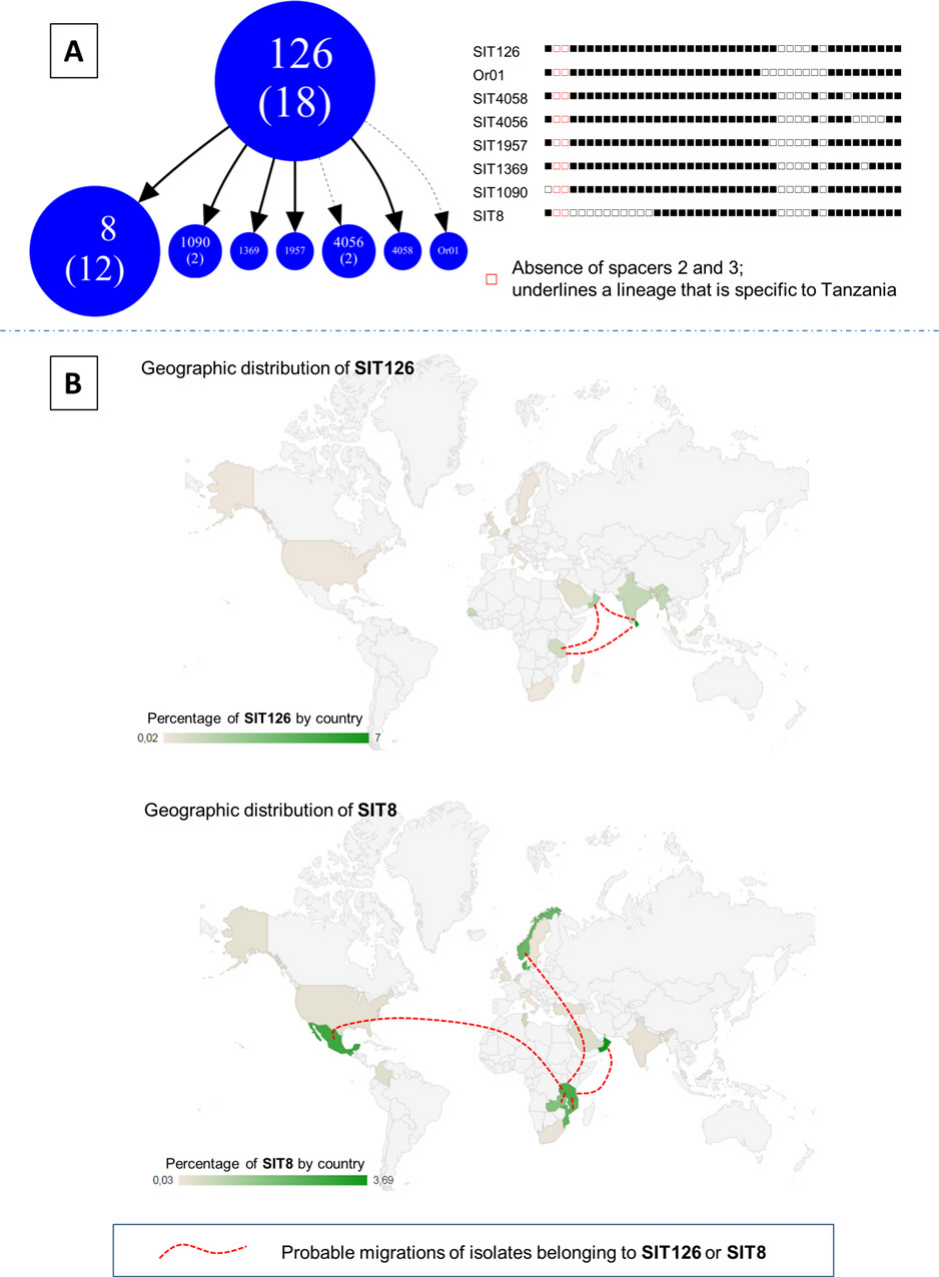
(**A**) Zoomed part of Spoligoforest tree showing SIT126 tentatively relabeled "EAI3-TZA" and its spoligotype descendants, along with their binary spoligotyping descriptions highlighting the specific absence of spacers 37, 38 and 40; and (**B**) Geographic distribution maps/Intensity maps of SIT126 and SIT8 (by percentage in country) according to SITVIT2 database before entering this study.

The analysis of spoligotype patterns from Tanzania based on the absence of specific spacers is shown in Fig 3A. Absence of spacers 2 and 3 indicated an EAI sublineage that seemed to be specific to Tanzania (pending further investigations). In total, there were 18 isolates belonging to SIT126 and 12 isolates belonging to SIT8. The peculiarity of SIT126 led to tentative re-labeling for the lineage as "EAI3-TZA" to indicate its specificity to Tanzania. This strain seemed to dominate in areas south of Asia particularly the Indian sub-continent (India and Sri-Lanka), Middle East (Oman) and Tanzania. The SIT8 lineage seems to be prevalent in Northern Europe (particularly in Denmark and Norway), Middle East (particularly in Oman), Eastern and Central Africa (particularly in Mozambique and Zambia) as well as southern part of North America particularly in the Mexican area. The geographic distribution maps/Intensity maps of SIT126 and SIT8 (by percentage in country, Fig 3B) has been established according to data recorded in the SITVIT2 database.

## Discussion

### General spoligotyping results

The present study reports for the first time, the results of mapping of MTBC in Tanzania and other African countries. The study combined spoligotyping data from Dar es Salaam city along the coast and the Serengeti ecosystem that comprises of Bunda, Serengeti and Ngorongoro with heterogeneous population variably involved in different activities as source of their income. Dar es Salaam and Bunda are the busiest cities due to their location that connects several other parts of Tanzania. The distribution of lineages/sublineages of isolates from Tanzania is shown in Table 1. Generally, our findings reveal similar spoligotyping results to those previously reported [13, 14, 16] with the exception that, a new EAI3 strain that seems to be specific to Tanzania was identified. We have tentatively re-labeled this sublineage as "EAI3-TZA” with reference to an international genotyping database SITVIT2, which is an updated version of previously released SITVITWEB database [26].

### TB and HIV Status

HIV infection has long been associated with tuberculosis, and this co-infection exacerbates the outcome of co-diseases. As regards TB patients whose HIV serology was known (214 strains), the findings that HIV-positive accounted for 21 (9.8%) cases calls for a joint consideration in designing control strategies. Although there were no statistically significant differences when comparing individual lineages versus HIV serology of the patients (p = 0.103), the proportion of HIV-positive patients isolates associated with LAM lineage strains (n = 6/38 or 15.79%) provides clues of HIV-TB strain specificity with patients. Some reports have in principle indicated similar associations [31] while others not [14]. With the reported multi-drug resistance in some areas by the LAM lineage [32], the finding is a warning for planning TB control strategy in HIV co-infection. It is important to note that significant number of T lineage (n = 7/57 or 12.28%) was also associated with HIV-positive patients. Despite variable reports on association between TB lineages and HIV [33–37], it seems most likely that there exists specific strain association with HIV. This association might be dominant strain dependent and probably region-specific. These all might be determined by transmission chains of infection. Furthermore, when comparing modern Euro-American PGG2/3 strains and ancestral PGG1 strains versus HIV serology, we found a significant difference. PGG2/3 strains were significantly more associated with HIV-positive patients as compared to PGG1 strains (p-value = 0.03; OR = 2.88, 95%CI [1.003–9.454]). The significant association found between PGG2/3 (modern) lineages group and HIV-positive patients is an observation that should provide some clues regarding treatment of co-infected TB/HIV cases that is mostly anticipated scenario presently. It could also mean that patients infected by evolutionary recent *M*. *tuberculosis* strains have a greater chance of reactivation from latent infection to disease than those infected with ancestral strains such as *M*. *africanum*.

The descriptive statistics on population characteristics reflected HIV serology distribution to not be significantly different between female and male patients (p-value = 0.931). The distribution was too, neither significant between the various cities of isolation (p-value = 0.921). The findings could mean that exposure to infection is more relevant and critical in establishing causal-impact relationship than disease-population characteristics interactions. Previous reports [1] have indicated the age range of 15–40 years, which is the most active and productive age, to be at a higher risk of TB infection than the rest. It is worth noting TB-HIV co-infection because either of the diseases exacerbate the impact or rather progression of the other. For example, it is said that the risk of progressing from latent to active TB in people living with HIV is 12 and 20 times greater than in those without HIV infection (www.who.int/tb/publications/global_report/). Despite the reported challenges in integrating TB and HIV control services (TB Facts.org, http://www.tbfacts.org/tb-hiv.html), it is ideal to have a conscious disease control approach whose focus is on TB-HIV co-infection in endemic areas. This is particularly critical when HIV infection impacts on the epidemiology of drug resistant TB. The idea is to reduce a high risk of development to a situation where MDR TB/HIV could become a co-epidemic [38] and worsening with drug resistant HIV infection. A combined TB/HIV vaccine that could include a BCG vaccine carrying combinations of both mycobacterial and HIV antigens [39, 40] has been suggested, however integrating the knowledge in the mechanistic interactive immune response in tuberculosis-HIV co-infection remains a challenge [40]. Yet, the current BCG vaccine does not confer effective and reliable protection against the prevalent pulmonary TB form in adults in countries near the equator.

### Distribution patterns of phylogenetical lineages

Interestingly, this study revealed a low prevalence (around 3%) of isolates belonging to the Manu family. Furthermore, a visible cleavage was noted for the lineage distribution in the studied cities (Fig 1). Bunda is a city located in the north-western part of the country near the Lake Victoria, as opposed to Dar es Salaam which is located in the eastern part of Tanzania. We noted that EAI lineage was more predominant in Bunda, Manu lineage was more associated to strains isolated in Ngorongoro, and CAS lineage was more predominant in Dar es Salaam (*p*-value<0.001); this observation may underline an agglomeration of EAI surrounding the Lake Victoria, but further investigations will be needed to clarify this fact as reflected by phylogeographical data.

The EAI strain is a highly polymorphic lineage [41] with considerably low transmission [42] that seem to emerge over 13,000 years ago (most recent common ancestor) having a markedly different genetic structure as compared to other human tuberculosis strains [43]. The strain belongs to the lineage 1 of the Indo-Oceanic family of TB strain [44] that include EAI-5, EAI1-SOM, EAI2-Manila, EAI2-Nonthaburi, EAI3-IND, EAI4-VNM, EAI6-BGD1, EAI7-BGD2, EAI8-MDG [45] in ancient classification [46] which is evolutionary primitive [47, 48]. Supposedly and proposed to have spread to East Africa from Asian continent (Southern India), predominance of this strain around the Lake zone might be due to its closeness to the Serengeti National Park. The CAS strain predominance in Dar es Salaam could be due to the city being very close to the Indian Ocean that links our country with The Asian continent. The CAS strain is said to emerge over 9,000 years ago [43] spreading globally. Using available genetic tools, spoligotyping and MIRU-VNTR, classification, surveillance on TB transmission trends and epidemiology have been made possible [30]. The CAS strain for example has been classified as belonging to the East-African Indian TB strain, lineage 3 according to other researchers [44]. In the SITVIT database, this lineage (labeled CAS) includes the CAS1-Delhi, CAS1-Kili, CAS2 and CAS (or CAS-like) [45]. Originally, while the EAI lineage is more ancestral, predominating in the southern part of India, the CAS lineage is predominant in the north of India and more modern [49]. The Manu lineage is a newly-described ancient clade, closely related to an Indo-Oceanic lineage 1 of an ancestral EAI strain of TB [44]. The presence of the Manu lineage in Ngorongoro could be a new introduction or a descendant of the EAI strain. The two lineages are closely related and have been proposed to share a common ancestor [49] or to represent an intermediate lineage between ancestral and evolutionary modern *M*. *tuberculosis* [50]. The presence of Manu lineage strains in the area could also be a warning sign as it has been reported to be associated with HIV infection [33].

### Tentative designation of EAI3-TZA as a new Tanzania-specific EAI sublineage

East Africa has been prone to nearly all TB strains from all over the world. Similar to other sub-Saharan Africa where HIV/AIDS is endemic, TB caseload increase 5x or more a decade in eastern and southern African countries [51]. This is partly attributed to the historical movements to and from Africa since colonial times particularly because movements were not strictly restricted. Studies done in different places in Asian continent have shown presence of confined local [52, 53] and predominant ancestral TB strains [54] whose worldwide spread might indicate their origins. It might signify that despite ancestral origins of TB strains there are microevolutions that are taking place within the MTBC lineages that give rise to new locally evolving strains that share specific signatures [53]. Evolution of *M*. *tuberculosis* strains analyzed using spoligotyping is generally through loss of some spacers. We understand that evolution is part of adaptation but it is still not known whether this evolution contributes to the ‘host-pathogen compatibility’ that was previously reported by Gagneux et al [44]. Despite the limited diversity in *M*. *tuberculosis* compared to other infectious bacteria [55], evolution in *M*. *tuberculosis* complex strains may result into new largely strain and lineage-specific characteristics that can influence the host interaction and pathogen virulence, consequently, determining the pathological potential and sequelae of infection [56].

Furthermore, a great proportion of SIT126/EAI5 was collected for the first time. The particular spoligotyping signature of this profile (absence of spacers 2 and 3) could indicate that it is endemic to East Africa (and particularly Tanzania). This SIT126 could also be a precursor of EAI3-IND and EAI8-MDG sublineages. We could hereby suggest a new EAI sublineage, tentatively designated as EAI3-TZA, which would be specific to Tanzania, and being mostly represented by SIT126 and its descendant SITs (Fig 3). This lineage which was initially proposed to be ‘Serengeti strains’ [15], seems to be not only spread in Serengeti ecosystem but also in other areas of Tanzania and possibly nearby countries. The lineage appeared to have spread in South India, Sri Lanka, and other Southeast Asian countries like Malaysia. This spread could be explained by ancestral Afro-Asian trade networks that occurred a long time ago (http://castinet.castilleja.org/users/pmckee/africaweb/swahilistates.html).

This specific family may have spread to other countries, bordering the Indian Ocean, due to historical trade reason or common history, like Oman which has historical links with Tanzania via Zanzibar (http://fr.wikipedia.org/wiki/Oman). However, the relevant proportion of SIT8 detected in Mexico could raise other questions. Several potential migration routes such as the route from East Africa, Middle East, South Asia, East Asia, North Asia, to the Americas by the Bering Strait, could explain the presence of SIT8 in Mexico. Nonetheless, more investigations are obviously needed to assert this hypothesis. Moreover, the large absence of spacers 2–13 in SIT8 does not allow us to draw significant conclusions on this profile. There might also be a parallel evolution that is taking place within *M*. *tuberculosis* complex providing another arm of consideration for the varying TB strain profiles with regions.

### Varying distribution patterns of Mycobacterium tuberculosis lineages in African countries

Variation in predominance of phylogenetic lineages in neighboring and other countries in Africa (Fig 1) largely reflect different ancestral origins of TB strains in the continent. It may also indicate diversity in global TB strains [57]. We noticed that the CAS family was predominant in the north (Sudan) and Eastern region covering Ethiopia, Kenya, and Tanzania, and to a lesser proportion in Madagascar. This lineage is said to descent from Central and Middle Eastern Asia consisting of relatively newly defined sub-lineages CAS1 and CAS2 [58]. Similarly, the EAI lineage was mostly present in Mozambique, Malawi, Sudan, Tanzania and Madagascar, and to a lesser extent in Kenya, Uganda, Zambia, Zimbabwe and Ethiopia. These strains are originally Asian particularly, Central Asia [49] and the region covered nears the Eastern route to and from Asia (Fig 3B). On the other hand, the predominance of LAM lineage was notable in Namibia, Zimbabwe, Zambia, Malawi, Mozambique, South Africa, Kenya, Tanzania and Madagascar. This strain seemed to largely predominate in the south and western part of Africa (Fig 1) that nears the South American-African migratory routes (Fig 3B). This might indicate that these strains originally had focal points of landing in Africa and later spreading within Africa due to movements within the continent. We can suggest that while strains like LAM are spreading from the South towards north along the eastern region, strains such as CAS, EAI, and Beijing counter spread (Fig 1). The T family spread is tremendously notable in most of the areas where TB data in Africa are available. This strain might largely signal evolutional changes that are occurring in the MTBC [53].

The varying predominance of other minor TB strains in Africa could be a result of intermitted migration in and out of Africa due to continued previous and recent movements. For example, the Manu family, which was also detected and reported in our study, could be originating from the north as it seems to prevail in Sudan and Uganda. The dynamics of lineage composition may depend on different selection pressures [59]. The X and S family seemed to be in limited distribution across African countries. As it was noted in Fig 1, unknown TB strains were detected in the majority of studied countries. These strains might be newly introduced or evolving strains. The other strains, CAM, TUR, AFRI and BOV which were reported in other African countries, were not detected in our Tanzanian study further signifying the localization and originality of various TB strains. It could be possible that, evolutionally, these various pathogens have been adapted to specific human populations [60]. The Beijing strain for instance is said to be very successful, virulent and potentially posing high risk for development of anti-TB drug resistance and multidrug resistance. This strain has an Asian ancestry with a geographical origin centered in northern China, Korea and Japan spreading out in waves [61] to other areas around the world. The worldwide spread of the Beijing lineage, raise concern on spread of drug resistance due to reported association with drug as well as multi-drug resistance [62–64]. With evolution, new strains can be expected. The newly emerging strains are usually more favoured than their ancestors making evolution a useful adaptation tool for the pathogen. New strains of EAI lineage have been reported elsewhere [65–67] indicating a continuum of evolution within this lineage. Similarly, in this study, we found a new EAI strain that does not look like other reported strains and it was tentatively renamed EAI-TZA.

In conclusion, this study provided mapping of MTBC genetic diversity in Tanzania (containing information on isolates from different cities) and several neighbouring African countries allowing us to underline potentially new spoligotyping patterns. However further genotyping information, such as MIRU-VNTRs typing, would be needed to pinpoint epidemiologically important clusters, which is the next important step in devising appropriate TB control strategy in Tanzania.

## Supporting Information

S1 Fig Spoligoforest tree drawn using the SpolTools software (available through http://www.emi.unsw.edu.au/spolTools; Reyes et al. [27], Tang et al. [26]), and shown as a Hierarchical Layout. The Figure was drawn on all patterns including orphan patterns (n = 293). Each spoligotype pattern from the study is represented by a node with area size being proportional to the total number of isolates with that specific pattern. Changes (loss of spacers) are represented by directed edges between nodes, with the arrowheads pointing to descendant spoligotypes. In this representation, the heuristic used selects a single inbound edge with a maximum weight using a Zipf model. Solid black lines link patterns that are very similar, i.e., loss of one spacer only (maximum weight being 1.0), while dashed lines represent links of weight comprised between 0.5 and 1, and dotted lines a weight less than 0.5.
(PDF)

S1 Table Detailed demographic, epidemiologic and genotyping information on Tanzanian M. tuberculosis isolates. Note that all strains were pansusceptible, and were isolated from newly diagnosed, sputum smear/culture positive pulmonary TB patients. NEW SITs are followed by an asterisk (*) and highlighted in yellow. Orphan spoligotypes are highlighted in blue.
(PDF)
