# Supplementary figures and images for: Mapping of Mycobacterium tuberculosis Complex Genetic Diversity Profiles in Tanzania and Other African Countries
Source: PLoS One. 2016 May 5;11(5):e0154571. doi: 10.1371/journal.pone.0154571 (PMC4858144; doi:10.1371/journal.pone.0154571)

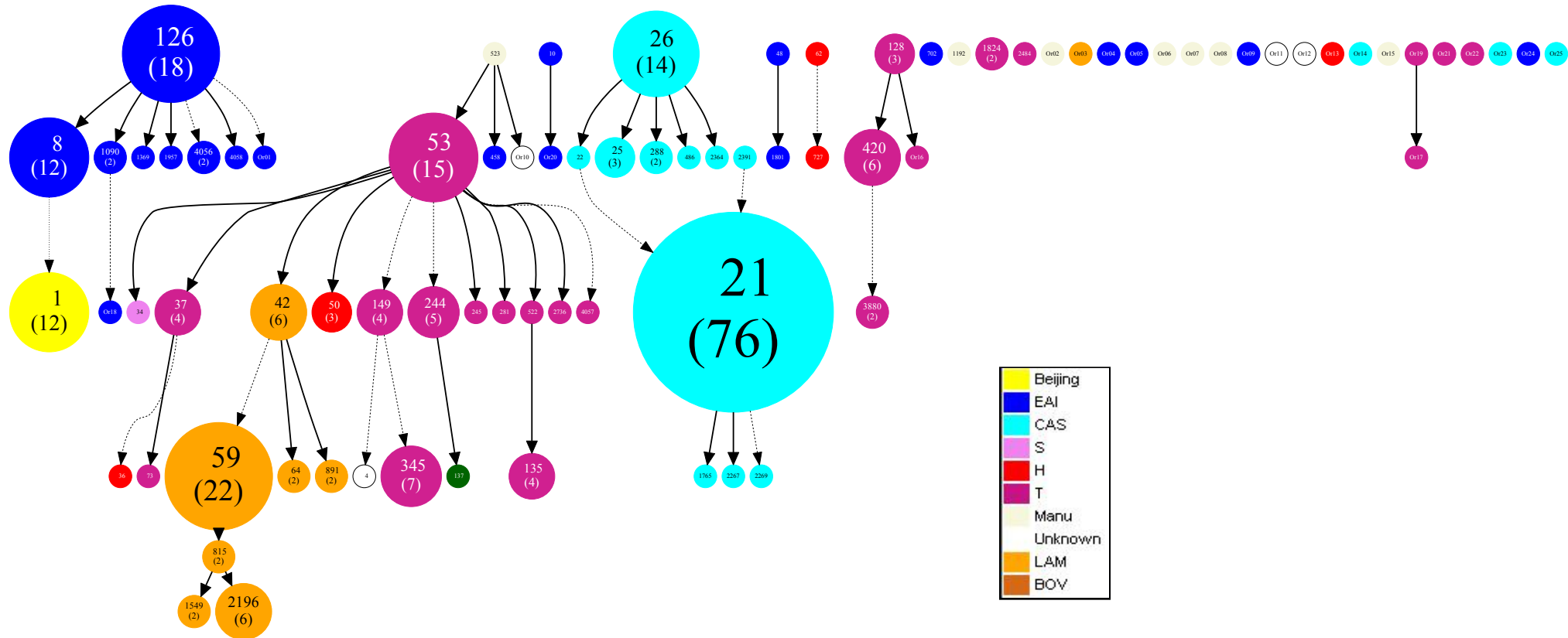

Supplement: S1 Fig — The Figure was drawn on all patterns including orphan patterns (n = 293). Each spoligotype pattern from the study is represented by a node with area size being proportional to the total number of isolates with that specific pattern. Changes (loss of spacers) are represented by directed edges between nodes, with the arrowheads pointing to descendant spoligotypes. In this representation, the heuristic used selects a single inbound edge with a maximum weight using a Zipf model. Solid black lines link patterns that are very similar, i.e., loss of one spacer only (maximum weight being 1.0), while dashed lines represent links of weight comprised between 0.5 and 1, and dotted lines a weight less than 0.5. (PDF) [file pone.0154571.s001.pdf]
