# Supplementary material for: Mapping of Mycobacterium tuberculosis Complex Genetic Diversity Profiles in Tanzania and Other African Countries
Source: PLoS One. 2016 May 5;11(5):e0154571. doi: 10.1371/journal.pone.0154571 (PMC4858144; doi:10.1371/journal.pone.0154571)
Supplement: S1 Table — Note that all strains were pansusceptible, and were isolated from newly diagnosed, sputum smear/culture positive pulmonary TB patients. NEW SITs are followed by an asterisk (*) and highlighted in yellow. Orphan spoligotypes are highlighted in blue. (PDF) [file pone.0154571.s002.pdf]

IsoNumber	Year	Strain	Spoligotype Description	Octal code	SIT	Lineage	Isolation city	Sex/Age	HIV Status
TZA0220121B0725	2012	B0725	□□□□□□□□□□□□□□□□□□□□□□□□□□□□□□□□□□■■■■■■■■■	000000000003771	1	Beijing	Bunda	F/41	positive
TZA0220101B0656	2010	B0656	□□□□□□□□□□□□□□□□□□□□□□□□□□□□□□□□□□■■■■■■■■■	000000000003771	1	Beijing	Bunda	M/31	negative
TZA0220111S0697	2011	S0697	□□□□□□□□□□□□□□□□□□□□□□□□□□□□□□□□□□■■■■■■■■■	000000000003771	1	Beijing	Serengeti	M/33	negative
TZA0220111S0705	2011	S0705	□□□□□□□□□□□□□□□□□□□□□□□□□□□□□□□□□□■■■■■■■■■	000000000003771	1	Beijing	Serengeti	M/22	negative
TZA0220111S0710	2011	S0710	□□□□□□□□□□□□□□□□□□□□□□□□□□□□□□□□□□■■■■■■■■■	000000000003771	1	Beijing	Serengeti	M/46	negative
TZA0220121S0700	2012	S0700	□□□□□□□□□□□□□□□□□□□□□□□□□□□□□□□□□□■■■■■■■■■	000000000003771	1	Beijing	Serengeti	M/51	negative
TZA0220121B0724	2012	B0724	□□□□□□□□□□□□□□□□□□□□□□□□□□□□□□□□□□■■■■■■■■■	000000000003771	1	Beijing	Bunda	M/37	negative
TZA0220121N1254	2012	N1254	□□□□□□□□□□□□□□□□□□□□□□□□□□□□□□□□□□■■■■■■■■■	000000000003771	1	Beijing	Ngorongoro	F/29	negative
TZA0220121D0167	2012	D0167	□□□□□□□□□□□□□□□□□□□□□□□□□□□□□□□□□□■■■■■■■■■	000000000003771	1	Beijing	Dar es Salaam	M/19	Not recorded
TZA0220111D0122	2011	D0122	□□□□□□□□□□□□□□□□□□□□□□□□□□□□□□□□□□■■■■■■■■■	000000000003771	1	Beijing	Dar es Salaam	M/27	Not recorded
TZA0220121D0150	2012	D0150	□□□□□□□□□□□□□□□□□□□□□□□□□□□□□□□□□□■■■■■■■■■	000000000003771	1	Beijing	Dar es Salaam	M/34	Not recorded
TZA0220111D0106	2011	D0106	□□□□□□□□□□□□□□□□□□□□□□□□□□□□□□□□□□■■■■■■■■■	000000000003771	1	Beijing	Dar es Salaam	F/43	Not recorded
TZA0220121N1012	2012	N1012	□□□□□□□□□□□□□□□□□□□□□□□□■■■■■■■■□□□□■■■■■■■	000000007760771	4	Unknown	Ngorongoro	F/25	negative
TZA0220111B0721	2011	B0721	■□□□□□□□□□□□□□□□■■■■■■■■■■■■■■■□□□□■□■■■■■■■■■	400037777413771	8	EAI5	Bunda	M/36	negative
TZA0220111N1273	2011	N1273	■□□□□□□□□□□□□□□□■■■■■■■■■■■■■■■□□□□■□■■■■■■■■■	400037777413771	8	EAI5	Ngorongoro	F/45	negative
TZA0220101B0587	2010	B0587	■□□□□□□□□□□□□□□□■■■■■■■■■■■■■■■□□□□■□■■■■■■■■■	400037777413771	8	EAI5	Bunda	F/28	positive
TZA0220101S0012	2010	S0012	■□□□□□□□□□□□□□□□■■■■■■■■■■■■■■■□□□□■□■■■■■■■■■	400037777413771	8	EAI5	Serengeti	F/25	negative
TZA0220121D0155	2012	D0155	■□□□□□□□□□□□□□□□■■■■■■■■■■■■■■■□□□□■□■■■■■■■■■	400037777413771	8	EAI5	Dar es Salaam	F/41	Not recorded
TZA0220121D0173	2012	D0173	■□□□□□□□□□□□□□□□■■■■■■■■■■■■■■■□□□□■□■■■■■■■■■	400037777413771	8	EAI5	Dar es Salaam	M/33	Not recorded
TZA0220121D0179	2012	D0179	■□□□□□□□□□□□□□□□■■■■■■■■■■■■■■■□□□□■□■■■■■■■■■	400037777413771	8	EAI5	Dar es Salaam	F/39	Not recorded
TZA0220121D0181	2012	D0181	■□□□□□□□□□□□□□□□■■■■■■■■■■■■■■■□□□□■□■■■■■■■■■	400037777413771	8	EAI5	Dar es Salaam	F/20	Not recorded
TZA0220121D0142	2012	D0142	■□□□□□□□□□□□□□□□■■■■■■■■■■■■■■■□□□□■□■■■■■■■■■	400037777413771	8	EAI5	Dar es Salaam	M/35	Not recorded
TZA0220121D0168	2012	D0168	■□□□□□□□□□□□□□□□■■■■■■■■■■■■■■■□□□□■□■■■■■■■■■	400037777413771	8	EAI5	Dar es Salaam	M/32	Not recorded
TZA0220111D0127	2011	D0127	■□□□□□□□□□□□□□□□■■■■■■■■■■■■■■■□□□□■□■■■■■■■■■	400037777413771	8	EAI5	Dar es Salaam	F/39	Not recorded
TZA0220111D0116	2011	D0116	■□□□□□□□□□□□□□□□■■■■■■■■■■■■■■■□□□□■□■■■■■■■■■	400037777413771	8	EAI5	Dar es Salaam	M/27	Not recorded
TZA0220111D0115	2011	D0115	■□□■■■■■■■■■■■■■■■□■□■■■■■■■□□□□■□■■■■■■■■■	477777277413771	10	EAI8-MDG	Dar es Salaam	M/26	Not recorded
TZA0220111N1172	2011	N1172	■■■□□□□■■□■■■■■■■■■□□□□□□□□□□□□□□□□■■■■■■■■	703377400001771	21	CAS1-Kili	Ngorongoro	M/42	negative
TZA0220101B0586	2010	B0586	■■■□□□□■■□■■■■■■■■■□□□□□□□□□□□□□□□□■■■■■■■■	703377400001771	21	CAS1-Kili	Bunda	F/38	negative
TZA0220101N0481	2010	N0481	■■■□□□□■■□■■■■■■■■■□□□□□□□□□□□□□□□□■■■■■■■■	703377400001771	21	CAS1-Kili	Ngorongoro	M/39	negative
TZA0220101N1265	2010	N1265	■■■□□□□■■□■■■■■■■■■□□□□□□□□□□□□□□□□■■■■■■■■	703377400001771	21	CAS1-Kili	Ngorongoro	M/32	negative
TZA0220101N1161	2010	N1161	■■■□□□□■■□■■■■■■■■■□□□□□□□□□□□□□□□□■■■■■■■■	703377400001771	21	CAS1-Kili	Ngorongoro	F/35	negative
TZA0220111B0588	2011	B0588	■■■□□□□■■□■■■■■■■■■□□□□□□□□□□□□□□□□■■■■■■■■	703377400001771	21	CAS1-Kili	Bunda	M/33	negative
TZA0220121N1175	2012	N1175	■■■□□□□■■□■■■■■■■■■□□□□□□□□□□□□□□□□■■■■■■■■	703377400001771	21	CAS1-Kili	Ngorongoro	M/38	negative
TZA0220101B0368	2010	B0368	■■■□□□□■■□■■■■■■■■■□□□□□□□□□□□□□□□□■■■■■■■■	703377400001771	21	CAS1-Kili	Bunda	M/31	negative
TZA0220121B0727	2012	B0727	■■■□□□□■■□■■■■■■■■■□□□□□□□□□□□□□□□□■■■■■■■■	703377400001771	21	CAS1-Kili	Bunda	F/43	negative
TZA0220101N0016	2010	N0016	■■■□□□□■■□■■■■■■■■■□□□□□□□□□□□□□□□□■■■■■■■■	703377400001771	21	CAS1-Kili	Ngorongoro	F/60	negative
TZA0220121N1010	2012	N1010	■■■□□□□■■□■■■■■■■■■□□□□□□□□□□□□□□□□■■■■■■■■	703377400001771	21	CAS1-Kili	Ngorongoro	M/44	positive
TZA0220121N1116	2012	N1116	■■■□□□□■■□■■■■■■■■■□□□□□□□□□□□□□□□□■■■■■■■■	703377400001771	21	CAS1-Kili	Ngorongoro	M/29	negative
TZA0220101N010-2	2010	N010-2	■■■□□□□■■□■■■■■■■■■□□□□□□□□□□□□□□□□■■■■■■■■	703377400001771	21	CAS1-Kili	Ngorongoro	M/51	negative
TZA0220101N010-5	2010	N010-5	■■■□□□□■■□■■■■■■■■■□□□□□□□□□□□□□□□□■■■■■■■■	703377400001771	21	CAS1-Kili	Ngorongoro	F/39	negative
TZA0220121N1014	2012	N1014	■■■□□□□■■□■■■■■■■■■□□□□□□□□□□□□□□□□■■■■■■■■	703377400001771	21	CAS1-Kili	Ngorongoro	F/37	negative
TZA0220111B0572	2011	B0572	■■■□□□□■■□■■■■■■■■■□□□□□□□□□□□□□□□□■■■■■■■■	703377400001771	21	CAS1-Kili	Bunda	F/47	negative
TZA0220121N1115	2012	N1115	■■■□□□□■■□■■■■■■■■■□□□□□□□□□□□□□□□□■■■■■■■■	703377400001771	21	CAS1-Kili	Ngorongoro	M/55	negative
TZA0220121N1131	2012	N1131	■■■□□□□■■□■■■■■■■■■□□□□□□□□□□□□□□□□■■■■■■■■	703377400001771	21	CAS1-Kili	Ngorongoro	M/52	negative
TZA0220101N1128	2010	N1128	■■■□□□□■■□■■■■■■■■■□□□□□□□□□□□□□□□□■■■■■■■■	703377400001771	21	CAS1-Kili	Ngorongoro	F/28	negative
TZA0220121N1130	2012	N1130	■■■□□□□■■□■■■■■■■■■□□□□□□□□□□□□□□□□■■■■■■■■	703377400001771	21	CAS1-Kili	Ngorongoro	M/47	negative
TZA0220121N1126	2012	N1126	■■■□□□□■■□■■■■■■■■■□□□□□□□□□□□□□□□□■■■■■■■■	703377400001771	21	CAS1-Kili	Ngorongoro	M/52	negative
TZA0220121N1127	2012	N1127	■■■□□□□■■□■■■■■■■■■□□□□□□□□□□□□□□□□■■■■■■■■	703377400001771	21	CAS1-Kili	Ngorongoro	M/45	negative
TZA0220111N1117	2011	N1117	■■■□□□□■■□■■■■■■■■■□□□□□□□□□□□□□□□□■■■■■■■■	703377400001771	21	CAS1-Kili	Ngorongoro	F/31	negative
TZA0220121N1124	2012	N1124	■■■□□□□■■□■■■■■■■■■□□□□□□□□□□□□□□□□■■■■■■■■	703377400001771	21	CAS1-Kili	Ngorongoro	F/33	positive
TZA0220121N1137	2012	N1137	■■■□□□□■■□■■■■■■■■■□□□□□□□□□□□□□□□□■■■■■■■■	703377400001771	21	CAS1-Kili	Ngorongoro	F/24	negative
TZA0220111N1118	2011	N1118	■■■□□□□■■□■■■■■■■■■□□□□□□□□□□□□□□□□■■■■■■■■	703377400001771	21	CAS1-Kili	Ngorongoro	M/28	negative
TZA0220111N1119	2011	N1119	■■■□□□□■■□■■■■■■■■■□□□□□□□□□□□□□□□□■■■■■■■■	703377400001771	21	CAS1-Kili	Ngorongoro	F/37	negative
TZA0220101N0487	2010	N0487	■■■□□□□■■□■■■■■■■■■□□□□□□□□□□□□□□□□■■■■■■■■	703377400001771	21	CAS1-Kili	Ngorongoro	F/25	negative
TZA0220121S0703	2012	S0703	■■■□□□□■■□■■■■■■■■■□□□□□□□□□□□□□□□□■■■■■■■■	703377400001771	21	CAS1-Kili	Serengeti	M/46	negative
TZA0220111S0709	2011	S0709	■■■□□□□■■□■■■■■■■■■□□□□□□□□□□□□□□□□■■■■■■■■	703377400001771	21	CAS1-Kili	Serengeti	F/37	negative
TZA0220111S0698	2011	S0698	■■■□□□□■■□■■■■■■■■■□□□□□□□□□□□□□□□□■■■■■■■■	703377400001771	21	CAS1-Kili	Serengeti	M/30	negative
TZA0220101N0485_b	2010	N0485_b	■■■□□□□■■□■■■■■■■■■□□□□□□□□□□□□□□□□■■■■■■■■	703377400001771	21	CAS1-Kili	Ngorongoro	F/30	negative
TZA0220111B0594	2011	B0594	■■■□□□□■■□■■■■■■■■■□□□□□□□□□□□□□□□□■■■■■■■■	703377400001771	21	CAS1-Kili	Bunda	F/39	negative

TZA0220101B0370_a	2010	B0370_a	■■■□□□□■■□■■■■■■■■■□□□□□□□□□□□□□□□□■■■■■■■■	703377400001771	21	CAS1-Kili	Bunda	F/24	negative
TZA0220111S0655	2011	S0655	■■■□□□□■■□■■■■■■■■■□□□□□□□□□□□□□□□□■■■■■■■■	703377400001771	21	CAS1-Kili	Serengeti	M/38	negative
TZA0220111B0513	2011	B0513	■■■□□□□■■□■■■■■■■■■□□□□□□□□□□□□□□□□■■■■■■■■	703377400001771	21	CAS1-Kili	Bunda	M/28	negative
TZA0220101N0482	2010	N0482	■■■□□□□■■□■■■■■■■■■□□□□□□□□□□□□□□□□■■■■■■■■	703377400001771	21	CAS1-Kili	Ngorongoro	F/39	negative
TZA0220101B0370_b	2010	B0370_b	■■■□□□□■■□■■■■■■■■■□□□□□□□□□□□□□□□□■■■■■■■■	703377400001771	21	CAS1-Kili	Bunda	M/35	negative
TZA0220121N1252	2012	N1252	■■■□□□□■■□■■■■■■■■■□□□□□□□□□□□□□□□□■■■■■■■■	703377400001771	21	CAS1-Kili	Ngorongoro	M/22	negative
TZA0220121D0175	2012	D0175	■■■□□□□■■□■■■■■■■■■□□□□□□□□□□□□□□□□■■■■■■■■	703377400001771	21	CAS1-Kili	Dar es Salaam	M/43	Not recorded
TZA0220121D0174	2012	D0174	■■■□□□□■■□■■■■■■■■■□□□□□□□□□□□□□□□□■■■■■■■■	703377400001771	21	CAS1-Kili	Dar es Salaam	F/37	Not recorded
TZA0220121D0161	2012	D0161	■■■□□□□■■□■■■■■■■■■□□□□□□□□□□□□□□□□■■■■■■■■	703377400001771	21	CAS1-Kili	Dar es Salaam	F/48	Not recorded
TZA0220121D0153	2012	D0153	■■■□□□□■■□■■■■■■■■■□□□□□□□□□□□□□□□□■■■■■■■■	703377400001771	21	CAS1-Kili	Dar es Salaam	F/45	Not recorded
TZA0220121D0162	2012	D0162	■■■□□□□■■□■■■■■■■■■□□□□□□□□□□□□□□□□■■■■■■■■	703377400001771	21	CAS1-Kili	Dar es Salaam	F/27	Not recorded
TZA0220121D0172	2012	D0172	■■■□□□□■■□■■■■■■■■■□□□□□□□□□□□□□□□□■■■■■■■■	703377400001771	21	CAS1-Kili	Dar es Salaam	M/36	Not recorded
TZA0220121D0145	2012	D0145	■■■□□□□■■□■■■■■■■■■□□□□□□□□□□□□□□□□■■■■■■■■	703377400001771	21	CAS1-Kili	Dar es Salaam	M/42	Not recorded
TZA0220121D0170	2012	D0170	■■■□□□□■■□■■■■■■■■■□□□□□□□□□□□□□□□□■■■■■■■■	703377400001771	21	CAS1-Kili	Dar es Salaam	M/65	Not recorded
TZA0220111D0137	2011	D0137	■■■□□□□■■□■■■■■■■■■□□□□□□□□□□□□□□□□■■■■■■■■	703377400001771	21	CAS1-Kili	Dar es Salaam	M/22	Not recorded
TZA0220121D0166	2012	D0166	■■■□□□□■■□■■■■■■■■■□□□□□□□□□□□□□□□□■■■■■■■■	703377400001771	21	CAS1-Kili	Dar es Salaam	F/31	Not recorded
TZA0220121D0146	2012	D0146	■■■□□□□■■□■■■■■■■■■□□□□□□□□□□□□□□□□■■■■■■■■	703377400001771	21	CAS1-Kili	Dar es Salaam	M/24	Not recorded
TZA0220121D0148	2012	D0148	■■■□□□□■■□■■■■■■■■■□□□□□□□□□□□□□□□□■■■■■■■■	703377400001771	21	CAS1-Kili	Dar es Salaam	M/33	Not recorded
TZA0220121D0164	2012	D0164	■■■□□□□■■□■■■■■■■■■□□□□□□□□□□□□□□□□■■■■■■■■	703377400001771	21	CAS1-Kili	Dar es Salaam	F/25	Not recorded
TZA0220111D0113	2011	D0113	■■■□□□□■■□■■■■■■■■■□□□□□□□□□□□□□□□□■■■■■■■■	703377400001771	21	CAS1-Kili	Dar es Salaam	M/23	Not recorded
TZA0220121D0171	2012	D0171	■■■□□□□■■□■■■■■■■■■□□□□□□□□□□□□□□□□■■■■■■■■	703377400001771	21	CAS1-Kili	Dar es Salaam	F/21	Not recorded
TZA0220121D1100	2012	D1100	■■■□□□□■■□■■■■■■■■■□□□□□□□□□□□□□□□□■■■■■■■■	703377400001771	21	CAS1-Kili	Dar es Salaam	M/27	Not recorded
TZA0220111D0126	2011	D0126	■■■□□□□■■□■■■■■■■■■□□□□□□□□□□□□□□□□■■■■■■■■	703377400001771	21	CAS1-Kili	Dar es Salaam	F/31	Not recorded
TZA0220111D0104	2011	D0104	■■■□□□□■■□■■■■■■■■■□□□□□□□□□□□□□□□□■■■■■■■■	703377400001771	21	CAS1-Kili	Dar es Salaam	F/30	Not recorded
TZA0220121D0160	2012	D0160	■■■□□□□■■□■■■■■■■■■□□□□□□□□□□□□□□□□■■■■■■■■	703377400001771	21	CAS1-Kili	Dar es Salaam	F/27	Not recorded
TZA0220121D0141	2012	D0141	■■■□□□□■■□■■■■■■■■■□□□□□□□□□□□□□□□□■■■■■■■■	703377400001771	21	CAS1-Kili	Dar es Salaam	M/39	Not recorded
TZA0220111D0136	2011	D0136	■■■□□□□■■□■■■■■■■■■□□□□□□□□□□□□□□□□■■■■■■■■	703377400001771	21	CAS1-Kili	Dar es Salaam	F/46	Not recorded
TZA0220111D0120	2011	D0120	■■■□□□□■■□■■■■■■■■■□□□□□□□□□□□□□□□□■■■■■■■■	703377400001771	21	CAS1-Kili	Dar es Salaam	M/23	Not recorded
TZA0220111D0131	2011	D0131	■■■□□□□■■□■■■■■■■■■□□□□□□□□□□□□□□□□■■■■■■■■	703377400001771	21	CAS1-Kili	Dar es Salaam	F/49	Not recorded
TZA0220111D0128	2011	D0128	■■■□□□□■■□■■■■■■■■■□□□□□□□□□□□□□□□□■■■■■■■■	703377400001771	21	CAS1-Kili	Dar es Salaam	F/37	Not recorded
TZA0220121D0156	2012	D0156	■■■□□□□■■□■■■■■■■■■□□□□□□□□□□□□□□□□■■■■■■■■	703377400001771	21	CAS1-Kili	Dar es Salaam	F/23	Not recorded
TZA0220121D0151	2012	D0151	■■■□□□□■■□■■■■■■■■■□□□□□□□□□□□□□□□□■■■■■■■■	703377400001771	21	CAS1-Kili	Dar es Salaam	F/28	Not recorded
TZA0220121D0140	2012	D0140	■■■□□□□■■□■■■■■■■■■□□□□□□□□□□□□□□□□■■■■■■■■	703377400001771	21	CAS1-Kili	Dar es Salaam	F/39	Not recorded
TZA0220111D0125	2011	D0125	■■■□□□□■■□■■■■■■■■■□□□□□□□□□□□□□□□□■■■■■■■■	703377400001771	21	CAS1-Kili	Dar es Salaam	M/21	Not recorded
TZA0220111D0108	2011	D0108	■■■□□□□■■□■■■■■■■■■□□□□□□□□□□□□□□□□■■■■■■■■	703377400001771	21	CAS1-Kili	Dar es Salaam	M/42	Not recorded
TZA0220111D0105	2011	D0105	■■■□□□□■■□■■■■■■■■■□□□□□□□□□□□□□□□□■■■■■■■■	703377400001771	21	CAS1-Kili	Dar es Salaam	M/41	Not recorded
TZA0220111D0102	2011	D0102	■■■□□□□■■□■■■■■■■■■□□□□□□□□□□□□□□□□■■■■■■■■	703377400001771	21	CAS1-Kili	Dar es Salaam	F/62	Not recorded
TZA0220111D0112	2011	D0112	■■■□□□□■■□■■■■■■■■■□□□□□□□□□□□□□□□□■■■■■■■■	703377400001771	21	CAS1-Kili	Dar es Salaam	M/33	Not recorded
TZA0220111D0123	2011	D0123	■■■□□□□■■□■■■■■■■■■□□□□□□□□□□□□□□□□■■■■■■■■	703377400001771	21	CAS1-Kili	Dar es Salaam	M/49	Not recorded
TZA0220121D1110	2012	D1110	■■■□□□□■■□■■■■■■■■■□□□□□□□□□□□□□□□□■■■■■■■■	703377400001771	21	CAS1-Kili	Dar es Salaam	M/51	Not recorded
TZA0220111D0135	2011	D0135	■■■□□□□■■□■■■■■■■■■□□□□□□□□□□□□□□□□■■■■■■■■	703377400001771	21	CAS1-Kili	Dar es Salaam	M/23	Not recorded
TZA0220111D0124	2011	D0124	■■■□□□□■■□■■■■■■■■■□□□□□□□□□□□□□□□□■■■■■■■■	703377400001771	21	CAS1-Kili	Dar es Salaam	F/35	Not recorded
TZA0220121D0165	2012	D0165	■■■□□□□■■□■■■■■■■■■□□□□□□□□□□□□□□□□■■■■■■■■	703377400001771	21	CAS1-Kili	Dar es Salaam	M/39	Not recorded
TZA0220121D0177	2012	D0177	■■■□□□□■■■■■■■■■■■■□□□□□□□□□□□□□□□□■■■■■■■■	703777400001771	22	CAS1-Delhi	Dar es Salaam	M/22	Not recorded
TZA0220111N1144	2011	N1144	■■■□□□□■■■■■■■■■■■■■■■□□□□□□□□□□□□■■□□■■■■■	703777740003171	25	CAS1-Delhi	Ngorongoro	M/52	positive
TZA0220121N1367	2012	N1367	■■■□□□□■■■■■■■■■■■■■■■□□□□□□□□□□□□■■□□■■■■■	703777740003171	25	CAS1-Delhi	Ngorongoro	M/32	negative
TZA0220111D0110	2011	D0110	■■■□□□□■■■■■■■■■■■■■■■□□□□□□□□□□□□■■□□■■■■■	703777740003171	25	CAS1-Delhi	Dar es Salaam	F/31	Not recorded
TZA0220101S0225	2010	S0225	■■■□□□□■■■■■■■■■■■■■■■□□□□□□□□□□□□■■■■■■■■■	703777740003771	26	CAS1-Delhi	Serengeti	F/22	negative
TZA0220111S0548	2011	S0548	■■■□□□□■■■■■■■■■■■■■■■□□□□□□□□□□□□■■■■■■■■■	703777740003771	26	CAS1-Delhi	Serengeti	F/27	negative
TZA0220101S0026_a	2010	S0026_a	■■■□□□□■■■■■■■■■■■■■■■□□□□□□□□□□□□■■■■■■■■■	703777740003771	26	CAS1-Delhi	Serengeti	M/54	negative
TZA0220111N1013	2011	N1013	■■■□□□□■■■■■■■■■■■■■■■□□□□□□□□□□□□■■■■■■■■■	703777740003771	26	CAS1-Delhi	Ngorongoro	F/27	negative
TZA0220121N1123	2012	N1123	■■■□□□□■■■■■■■■■■■■■■■□□□□□□□□□□□□■■■■■■■■■	703777740003771	26	CAS1-Delhi	Ngorongoro	M/42	negative
TZA0220101S0256	2010	S0256	■■■□□□□■■■■■■■■■■■■■■■□□□□□□□□□□□□■■■■■■■■■	703777740003771	26	CAS1-Delhi	Serengeti	M/38	negative
TZA0220111S0696	2011	S0696	■■■□□□□■■■■■■■■■■■■■■■□□□□□□□□□□□□■■■■■■■■■	703777740003771	26	CAS1-Delhi	Serengeti	F/37	negative
TZA0220111S0699	2011	S0699	■■■□□□□■■■■■■■■■■■■■■■□□□□□□□□□□□□■■■■■■■■■	703777740003771	26	CAS1-Delhi	Serengeti	F/35	negative
TZA0220121B0714_a	2012	B0714_a	■■■□□□□■■■■■■■■■■■■■■■□□□□□□□□□□□□■■■■■■■■■	703777740003771	26	CAS1-Delhi	Bunda	M/39	negative
TZA0220111S0707	2011	S0707	■■■□□□□■■■■■■■■■■■■■■■□□□□□□□□□□□□■■■■■■■■■	703777740003771	26	CAS1-Delhi	Serengeti	F/21	negative
TZA0220101S0026_b	2010	S0026_b	■■■□□□□■■■■■■■■■■■■■■■□□□□□□□□□□□□■■■■■■■■■	703777740003771	26	CAS1-Delhi	Serengeti	M/21	negative
TZA0220121D0176	2012	D0176	■■■□□□□■■■■■■■■■■■■■■■□□□□□□□□□□□□■■■■■■■■■	703777740003771	26	CAS1-Delhi	Dar es Salaam	M/21	Not recorded
TZA0220111D0133	2011	D0133	■■■□□□□■■■■■■■■■■■■■■■□□□□□□□□□□□□■■■■■■■■■	703777740003771	26	CAS1-Delhi	Dar es Salaam	F/38	Not recorded
TZA0220121D0154	2012	D0154	■■■□□□□■■■■■■■■■■■■■■■□□□□□□□□□□□□■■■■■■■■■	703777740003771	26	CAS1-Delhi	Dar es Salaam	M/75	Not recorded
TZA0220111B0510	2011	B0510	■■■■■■■■□□■■■■■■■■■■■■■■■■■■■■■■□□□□■■■■■■■	776377777760771	34	S	Bunda	F/41	positive
TZA0220101N0055	2010	N0055	■■■■■■■■■■■■□■■■■■■■■■■■■■■■■□■□□□□■■■■■■■	777737777720771	36	H3	Ngorongoro	M/39	negative

TZA0220111B0647	2011	B0647	■■■■■■■■■■■■□■■■■■■■■■■■■■■■■■■■■□□□□■■■■■■■	777737777760771	37	T3	Bunda	F/34	negative
TZA0220121B0646	2012	B0646	■■■■■■■■■■■■□■■■■■■■■■■■■■■■■■■■■□□□□■■■■■■■	777737777760771	37	T3	Bunda	F/33	negative
TZA0220111D0117	2011	D0117	■■■■■■■■■■■■□■■■■■■■■■■■■■■■■■■■■□□□□■■■■■■■	777737777760771	37	T3	Dar es Salaam	F/40	Not recorded
TZA0220111D0109	2011	D0109	■■■■■■■■■■■■□■■■■■■■■■■■■■■■■■■■■□□□□■■■■■■■	777737777760771	37	T3	Dar es Salaam	F/25	Not recorded
TZA0220111N1372	2011	N1372	■■■■■■■■■■■■■■■■■■■■□□□□■■■■■■■■□□□□■■■■■■■	777777607760771	42	LAM9	Ngorongoro	M/30	negative
TZA0220101N1256	2010	N1256	■■■■■■■■■■■■■■■■■■■■□□□□■■■■■■■■□□□□■■■■■■■	777777607760771	42	LAM9	Ngorongoro	M/22	positive
TZA0220111N1259	2011	N1259	■■■■■■■■■■■■■■■■■■■■□□□□■■■■■■■■□□□□■■■■■■■	777777607760771	42	LAM9	Ngorongoro	M/37	negative
TZA0220111N0584	2011	N0584	■■■■■■■■■■■■■■■■■■■■□□□□■■■■■■■■□□□□■■■■■■■	777777607760771	42	LAM9	Ngorongoro	M/34	negative
TZA0220121N1276	2012	N1276	■■■■■■■■■■■■■■■■■■■■□□□□■■■■■■■■□□□□■■■■■■■	777777607760771	42	LAM9	Ngorongoro	M/33	positive
TZA0220111D0130	2011	D0130	■■■■■■■■■■■■■■■■■■■■□□□□■■■■■■■■□□□□■■■■■■■	777777607760771	42	LAM9	Dar es Salaam	F/48	Not recorded
TZA0220121D0139	2012	D0139	■■■■■■■■■■■■■■■■■■■■■■■■■■■■□□□□■□■■■■■□■■■	777777777413731	48	EAI1-SOM	Dar es Salaam	M/30	Not recorded
TZA0220121S0663	2012	S0663	■■■■■■■■■■■■■■■■■■■■■■■■■■■■■■□■□□□□■■■■■■■	777777777720771	50	H3	Serengeti	M/35	negative
TZA0220111B0666	2011	B0666	■■■■■■■■■■■■■■■■■■■■■■■■■■■■■■□■□□□□■■■■■■■	777777777720771	50	H3	Bunda	F/45	negative
TZA0220111S0664	2011	S0664	■■■■■■■■■■■■■■■■■■■■■■■■■■■■■■□■□□□□■■■■■■■	777777777720771	50	H3	Serengeti	M/40	negative
TZA0220101B0385	2010	B0385	■■■■■■■■■■■■■■■■■■■■■■■■■■■■■■■■□□□□■■■■■■■	777777777760771	53	T1	Bunda	M/21	negative
TZA0220101B0133	2010	B0133	■■■■■■■■■■■■■■■■■■■■■■■■■■■■■■■■□□□□■■■■■■■	777777777760771	53	T1	Bunda	F/22	negative
TZA0220111B0581	2011	B0581	■■■■■■■■■■■■■■■■■■■■■■■■■■■■■■■■□□□□■■■■■■■	777777777760771	53	T1	Bunda	F/50	negative
TZA0220111B0577	2011	B0577	■■■■■■■■■■■■■■■■■■■■■■■■■■■■■■■■□□□□■■■■■■■	777777777760771	53	T1	Bunda	F/21	negative
TZA0220121N1136	2012	N1136	■■■■■■■■■■■■■■■■■■■■■■■■■■■■■■■■□□□□■■■■■■■	777777777760771	53	T1	Ngorongoro	F/32	negative
TZA0220101B0392	2010	B0392	■■■■■■■■■■■■■■■■■■■■■■■■■■■■■■■■□□□□■■■■■■■	777777777760771	53	T1	Bunda	M/41	negative
TZA0220111S0704	2011	S0704	■■■■■■■■■■■■■■■■■■■■■■■■■■■■■■■■□□□□■■■■■■■	777777777760771	53	T1	Serengeti	M/38	negative
TZA0220111B0693	2011	B0693	■■■■■■■■■■■■■■■■■■■■■■■■■■■■■■■■□□□□■■■■■■■	777777777760771	53	T1	Bunda	M/38	negative
TZA0220121B0714_b	2012	B0714_b	■■■■■■■■■■■■■■■■■■■■■■■■■■■■■■■■□□□□■■■■■■■	777777777760771	53	T1	Bunda	M/45	negative
TZA0220111B0582	2011	B0582	■■■■■■■■■■■■■■■■■■■■■■■■■■■■■■■■□□□□■■■■■■■	777777777760771	53	T1	Bunda	M/59	negative
TZA0220101B0391	2010	B0391	■■■■■■■■■■■■■■■■■■■■■■■■■■■■■■■■□□□□■■■■■■■	777777777760771	53	T1	Bunda	M/33	negative
TZA0220101B0387	2010	B0387	■■■■■■■■■■■■■■■■■■■■■■■■■■■■■■■■□□□□■■■■■■■	777777777760771	53	T1	Bunda	M/51	negative
TZA0220121D0169	2012	D0169	■■■■■■■■■■■■■■■■■■■■■■■■■■■■■■■■□□□□■■■■■■■	777777777760771	53	T1	Dar es Salaam	F/41	Not recorded
TZA0220111D0118	2011	D0118	■■■■■■■■■■■■■■■■■■■■■■■■■■■■■■■■□□□□■■■■■■■	777777777760771	53	T1	Dar es Salaam	M/34	Not recorded
TZA0220121D0157	2012	D0157	■■■■■■■■■■■■■■■■■■■■■■■■■■■■■■■■□□□□■■■■■■■	777777777760771	53	T1	Dar es Salaam	M/63	Not recorded
TZA0220111N1164	2011	N1164	■■■■■■■■■■■■■■■■■■■■□□□□■■□□□□■■□□□□■■■■■■■	777777606060771	59	LAM11-ZWE	Ngorongoro	M/21	negative
TZA0220111N1163	2011	N1163	■■■■■■■■■■■■■■■■■■■■□□□□■■□□□□■■□□□□■■■■■■■	777777606060771	59	LAM11-ZWE	Ngorongoro	F/40	positive
TZA0220111B0636	2011	B0636	■■■■■■■■■■■■■■■■■■■■□□□□■■□□□□■■□□□□■■■■■■■	777777606060771	59	LAM11-ZWE	Bunda	F/41	negative
TZA0220111B0695	2011	B0695	■■■■■■■■■■■■■■■■■■■■□□□□■■□□□□■■□□□□■■■■■■■	777777606060771	59	LAM11-ZWE	Bunda	M/51	negative
TZA0220121N1243	2012	N1243	■■■■■■■■■■■■■■■■■■■■□□□□■■□□□□■■□□□□■■■■■■■	777777606060771	59	LAM11-ZWE	Ngorongoro	F/20	positive
TZA0220101S0264	2010	S0264	■■■■■■■■■■■■■■■■■■■■□□□□■■□□□□■■□□□□■■■■■■■	777777606060771	59	LAM11-ZWE	Serengeti	M/40	negative
TZA0220101N0068	2010	N0068	■■■■■■■■■■■■■■■■■■■■□□□□■■□□□□■■□□□□■■■■■■■	777777606060771	59	LAM11-ZWE	Ngorongoro	F/25	negative
TZA0220101S0263	2010	S0263	■■■■■■■■■■■■■■■■■■■■□□□□■■□□□□■■□□□□■■■■■■■	777777606060771	59	LAM11-ZWE	Serengeti	F/33	negative
TZA0220101N010-1	2010	N010-1	■■■■■■■■■■■■■■■■■■■■□□□□■■□□□□■■□□□□■■■■■■■	777777606060771	59	LAM11-ZWE	Ngorongoro	F/45	negative
TZA0220101N010-3	2010	N010-3	■■■■■■■■■■■■■■■■■■■■□□□□■■□□□□■■□□□□■■■■■■■	777777606060771	59	LAM11-ZWE	Ngorongoro	M/53	negative
TZA0220121N1121	2012	N1121	■■■■■■■■■■■■■■■■■■■■□□□□■■□□□□■■□□□□■■■■■■■	777777606060771	59	LAM11-ZWE	Ngorongoro	F/34	negative
TZA0220121B0632	2012	B0632	■■■■■■■■■■■■■■■■■■■■□□□□■■□□□□■■□□□□■■■■■■■	777777606060771	59	LAM11-ZWE	Bunda	M/23	negative
TZA0220121B0631	2012	B0631	■■■■■■■■■■■■■■■■■■■■□□□□■■□□□□■■□□□□■■■■■■■	777777606060771	59	LAM11-ZWE	Bunda	M/32	negative
TZA0220121N1133	2012	N1133	■■■■■■■■■■■■■■■■■■■■□□□□■■□□□□■■□□□□■■■■■■■	777777606060771	59	LAM11-ZWE	Ngorongoro	F/40	negative
TZA0220101B0374_b	2010	B0374_b	■■■■■■■■■■■■■■■■■■■■□□□□■■□□□□■■□□□□■■■■■■■	777777606060771	59	LAM11-ZWE	Bunda	M/22	negative
TZA0220101B0375	2010	B0375	■■■■■■■■■■■■■■■■■■■■□□□□■■□□□□■■□□□□■■■■■■■	777777606060771	59	LAM11-ZWE	Bunda	F/24	negative
TZA0220101B0373	2010	B0373	■■■■■■■■■■■■■■■■■■■■□□□□■■□□□□■■□□□□■■■■■■■	777777606060771	59	LAM11-ZWE	Bunda	M/18	negative
TZA0220111B0686	2011	B0686	■■■■■■■■■■■■■■■■■■■■□□□□■■□□□□■■□□□□■■■■■■■	777777606060771	59	LAM11-ZWE	Bunda	F/35	negative
TZA0220121N1242	2012	N1242	■■■■■■■■■■■■■■■■■■■■□□□□■■□□□□■■□□□□■■■■■■■	777777606060771	59	LAM11-ZWE	Ngorongoro	F/27	negative
TZA0220121D0147	2012	D0147	■■■■■■■■■■■■■■■■■■■■□□□□■■□□□□■■□□□□■■■■■■■	777777606060771	59	LAM11-ZWE	Dar es Salaam	M/38	Not recorded
TZA0220111D0138	2011	D0138	■■■■■■■■■■■■■■■■■■■■□□□□■■□□□□■■□□□□■■■■■■■	777777606060771	59	LAM11-ZWE	Dar es Salaam	F/25	Not recorded
TZA0220121D0178	2012	D0178	■■■■■■■■■■■■■■■■■■■■□□□□■■□□□□■■□□□□■■■■■■■	777777606060771	59	LAM11-ZWE	Dar es Salaam	F/29	Not recorded
TZA0220121N1365	2012	N1365	■■■■■■■■■■■■■■■■■■■■■■■■■□□□□□□■□□□□■■■□■■■	777777774020731	62	H1	Ngorongoro	M/30	negative
TZA0220121N1249	2012	N1249	■■■■■■■■■■■■■■■■■■■■□□□□■■■■□■■■□□□□■■■■■■■	777777607560771	64	LAM6	Ngorongoro	M/39	negative
TZA0220111D0114	2011	D0114	■■■■■■■■■■■■■■■■■■■■□□□□■■■■□■■■□□□□■■■■■■■	777777607560771	64	LAM6	Dar es Salaam	M/36	Not recorded
TZA0220121N1120	2012	N1120	■■■■■■■■■■■■□■■■■■■■■■■■■■■■■■■■■□□□□■■■□■■■	777737777760731	73	T	Ngorongoro	M/29	negative
TZA0220101B0374_a	2010	B0374_a	■□□■■■■■■■■■■■■■■■■■■■■■■■■■□□□□■□■■■■■■■■■	477777777413771	126	EAI5	Bunda	M/41	negative
TZA0220121B0732	2012	B0732	■□□■■■■■■■■■■■■■■■■■■■■■■■■■□□□□■□■■■■■■■■■	477777777413771	126	EAI5	Bunda	F/21	negative
TZA0220111S0677	2011	S0677	■□□■■■■■■■■■■■■■■■■■■■■■■■■■□□□□■□■■■■■■■■■	477777777413771	126	EAI5	Serengeti	F/24	negative
TZA0220111B0613	2011	B0613	■□□■■■■■■■■■■■■■■■■■■■■■■■■■□□□□■□■■■■■■■■■	477777777413771	126	EAI5	Bunda	M/19	negative
TZA0220121N1122	2012	N1122	■□□■■■■■■■■■■■■■■■■■■■■■■■■■□□□□■□■■■■■■■■■	477777777413771	126	EAI5	Ngorongoro	F/40	negative
TZA0220101B0494	2010	B0494	■□□■■■■■■■■■■■■■■■■■■■■■■■■■□□□□■□■■■■■■■■■	477777777413771	126	EAI5	Bunda	M/38	negative
TZA0220111N0564	2011	N0564	■□□■■■■■■■■■■■■■■■■■■■■■■■■■□□□□■□■■■■■■■■■	477777777413771	126	EAI5	Ngorongoro	M/20	negative
TZA0220111B0379	2011	B0379	■□□■■■■■■■■■■■■■■■■■■■■■■■■■□□□□■□■■■■■■■■■	477777777413771	126	EAI5	Bunda	F/29	negative

TZA0220101B0384	2010	B0384	■□□■■■■■■■■■■■■■■■■■■■■■■■■■□□□□■□■■■■■■■■■	477777777413771	126	EAI5	Bunda	M/54	negative
TZA0220121N0589	2012	N0589	■□□■■■■■■■■■■■■■■■■■■■■■■■■■□□□□■□■■■■■■■■■	477777777413771	126	EAI5	Ngorongoro	F/25	negative
TZA0220111S0678	2011	S0678	■□□■■■■■■■■■■■■■■■■■■■■■■■■■□□□□■□■■■■■■■■■	477777777413771	126	EAI5	Serengeti	F/20	negative
TZA0220111B0675	2011	B0675	■□□■■■■■■■■■■■■■■■■■■■■■■■■■□□□□■□■■■■■■■■■	477777777413771	126	EAI5	Bunda	M/40	negative
TZA0220111S0676	2011	S0676	■□□■■■■■■■■■■■■■■■■■■■■■■■■■□□□□■□■■■■■■■■■	477777777413771	126	EAI5	Serengeti	F/25	negative
TZA0220101B0383	2010	B0383	■□□■■■■■■■■■■■■■■■■■■■■■■■■■□□□□■□■■■■■■■■■	477777777413771	126	EAI5	Bunda	F/30	negative
TZA0220101B0378	2010	B0378	■□□■■■■■■■■■■■■■■■■■■■■■■■■■□□□□■□■■■■■■■■■	477777777413771	126	EAI5	Bunda	F/27	negative
TZA0220101B0307	2010	B0307	■□□■■■■■■■■■■■■■■■■■■■■■■■■■□□□□■□■■■■■■■■■	477777777413771	126	EAI5	Bunda	F/35	negative
TZA0220121D0159	2012	D0159	■□□■■■■■■■■■■■■■■■■■■■■■■■■■□□□□■□■■■■■■■■■	477777777413771	126	EAI5	Dar es Salaam	F/50	Not recorded
TZA0220111D0144	2011	D0144	■□□■■■■■■■■■■■■■■■■■■■■■■■■■□□□□■□■■■■■■■■■	477777777413771	126	EAI5	Dar es Salaam	F/19	Not recorded
TZA0220101S0011_a	2010	S0011_a	■■□□■■■■■■■■■■■■□■■■■■■■■■■■■■■■□□□□■■■□■■□	637775777760730	128	T2-uganda	Serengeti	M/46	negative
TZA0220101S0011_b	2010	S0011_b	■■□□■■■■■■■■■■■■□■■■■■■■■■■■■■■■□□□□■■■□■■□	637775777760730	128	T2-uganda	Serengeti	F/39	negative
TZA0220111D0103	2011	D0103	■■□□■■■■■■■■■■■■□■■■■■■■■■■■■■■■□□□□■■■□■■□	637775777760730	128	T2-uganda	Dar es Salaam	M/38	Not recorded
TZA0220111S0684	2011	S0684	■■■■■■■■■■■■■■■■■■■■■■■■■■■■■■■■□□□□■■■□■■□	777777777760730	135	T2-uganda	Serengeti	M/39	positive
TZA0220111S0681	2011	S0681	■■■■■■■■■■■■■■■■■■■■■■■■■■■■■■■■□□□□■■■□■■□	777777777760730	135	T2-uganda	Serengeti	M/22	negative
TZA0220111B0679	2011	B0679	■■■■■■■■■■■■■■■■■■■■■■■■■■■■■■■■□□□□■■■□■■□	777777777760730	135	T2-uganda	Bunda	M/31	negative
TZA0220111S0683	2011	S0683	■■■■■■■■■■■■■■■■■■■■■■■■■■■■■■■■□□□□■■■□■■□	777777777760730	135	T2-uganda	Serengeti	F/28	negative
TZA0220101N0052	2010	N0052	■■■■■■■■■■■■■■■■■□■■■■■■■■■■■■■■□□□□■■□□□□■	777776777760601	137	X2	Ngorongoro	F/37	negative
TZA0220101B0652	2010	B0652	■■■■■■■■■□□□□□□□□□□■■■■■■■■■■■■■□□□□■■■■■■■	777000377760771	149	T3-ETH	Bunda	M/36	negative
TZA0220121B0715	2012	B0715	■■■■■■■■■□□□□□□□□□□■■■■■■■■■■■■■□□□□■■■■■■■	777000377760771	149	T3-ETH	Bunda	M/20	positive
TZA0220111B0712	2011	B0712	■■■■■■■■■□□□□□□□□□□■■■■■■■■■■■■■□□□□■■■■■■■	777000377760771	149	T3-ETH	Bunda	F/41	negative
TZA0220121B0716	2012	B0716	■■■■■■■■■□□□□□□□□□□■■■■■■■■■■■■■□□□□■■■■■■■	777000377760771	149	T3-ETH	Bunda	F/47	negative
TZA0220101N010-6	2010	N010-6	■■■■■■■■■■■■■■■■■■■■■■■■■■■■■■■■□□□□■■□□□□■	777777777760601	244	T1	Ngorongoro	M/36	negative
TZA0220111B0630	2011	B0630	■■■■■■■■■■■■■■■■■■■■■■■■■■■■■■■■□□□□■■□□□□■	777777777760601	244	T1	Bunda	M/49	negative
TZA0220101N0196	2010	N0196	■■■■■■■■■■■■■■■■■■■■■■■■■■■■■■■■□□□□■■□□□□■	777777777760601	244	T1	Ngorongoro	M/33	negative
TZA0220111N0419	2011	N0419	■■■■■■■■■■■■■■■■■■■■■■■■■■■■■■■■□□□□■■□□□□■	777777777760601	244	T1	Ngorongoro	F/29	negative
TZA0220101N0051	2010	N0051	■■■■■■■■■■■■■■■■■■■■■■■■■■■■■■■■□□□□■■□□□□■	777777777760601	244	T1	Ngorongoro	F/40	negative
TZA0220101N1239	2010	N1239	■■■■■■■■■■■■■■■■■■■■■■■■■■■■■■■■□□□□■■□■■■■	777777777760671	245	T1	Ngorongoro	F/29	negative
TZA0220111D0134	2011	D0134	■■■■■■■■■■■■■■■■□■■■■■■■■■■■■■■■□□□□■■■■■■■	777775777760771	281	T1	Dar es Salaam	M/39	Not recorded
TZA0220111B0603	2011	B0603	■■■□□□□□□□■■■■■■■■■■■■□□□□□□□□□□□□■■■■■■■■■	700377740003771	288	CAS2	Bunda	F/30	negative
TZA0220121D0149	2012	D0149	■■■□□□□□□□■■■■■■■■■■■■□□□□□□□□□□□□■■■■■■■■■	700377740003771	288	CAS2	Dar es Salaam	M/35	Not recorded
TZA0220111N0483	2011	N0483	■■■■■■■■■□□□□□□□□□□■■■■■■■■■■■■■□□□□■■■□■■■	777000377760731	345	T3-ETH	Ngorongoro	F/29	negative
TZA0220101N0484	2010	N0484	■■■■■■■■■□□□□□□□□□□■■■■■■■■■■■■■□□□□■■■□■■■	777000377760731	345	T3-ETH	Ngorongoro	F/41	negative
TZA0220111N0485_a	2011	N0485_a	■■■■■■■■■□□□□□□□□□□■■■■■■■■■■■■■□□□□■■■□■■■	777000377760731	345	T3-ETH	Ngorongoro	F/22	negative
TZA0220101N0486	2010	N0486	■■■■■■■■■□□□□□□□□□□■■■■■■■■■■■■■□□□□■■■□■■■	777000377760731	345	T3-ETH	Ngorongoro	F/20	negative
TZA0220121S0701	2012	S0701	■■■■■■■■■□□□□□□□□□□■■■■■■■■■■■■■□□□□■■■□■■■	777000377760731	345	T3-ETH	Serengeti	F/33	negative
TZA0220101N0479	2010	N0479	■■■■■■■■■□□□□□□□□□□■■■■■■■■■■■■■□□□□■■■□■■■	777000377760731	345	T3-ETH	Ngorongoro	M/21	negative
TZA0220111S0558	2011	S0558	■■■■■■■■■□□□□□□□□□□■■■■■■■■■■■■■□□□□■■■□■■■	777000377760731	345	T3-ETH	Serengeti	M/51	negative
TZA0220101S0005	2010	S0005	■■□□■■■■■■■■■■■■□□■■■■■■■■■■■■■■□□□□■■■□■■□	637774777760730	420	T2-uganda	Serengeti	M/30	negative
TZA0220101S0006	2010	S0006	■■□□■■■■■■■■■■■■□□■■■■■■■■■■■■■■□□□□■■■□■■□	637774777760730	420	T2-uganda	Serengeti	M/22	negative
TZA0220101S0010	2010	S0010	■■□□■■■■■■■■■■■■□□■■■■■■■■■■■■■■□□□□■■■□■■□	637774777760730	420	T2-uganda	Serengeti	M/35	negative
TZA0220101S0002_a	2010	S0002_a	■■□□■■■■■■■■■■■■□□■■■■■■■■■■■■■■□□□□■■■□■■□	637774777760730	420	T2-uganda	Serengeti	M/39	positive
TZA0220101S0009	2010	S0009	■■□□■■■■■■■■■■■■□□■■■■■■■■■■■■■■□□□□■■■□■■□	637774777760730	420	T2-uganda	Serengeti	F/34	negative
TZA0220101S0002_b	2010	S0002_b	■■□□■■■■■■■■■■■■□□■■■■■■■■■■■■■■□□□□■■■□■■□	637774777760730	420	T2-uganda	Serengeti	M/44	positive
TZA0220101B0057	2010	B0057	■■■■■■■■■■■■■■■■■■■■■■■■■■■■□□□□□□■■■■■■■■■	777777777403771	458	EAI5	Bunda	M/37	negative
TZA0220121D0143	2012	D0143	■■■□□□□■■■■■■■■■■■■■■■□□□□□□□□□□□□□□□■■■■■■	703777740000371	486	CAS	Dar es Salaam	M/51	Not recorded
TZA0220111B0576	2011	B0576	■■■■■■■■■■■■■■■■■■■■■■■■■■■■■■■■□□□□■■■■■■□	777777777760770	522	T1	Bunda	F/45	negative
TZA0220101N010-4	2010	N010-4	■■■	777777777777771	523	Manu_ancestor	Ngorongoro	M/22	negative
TZA0220121D0158	2012	D0158	■■■□□□□□□■■■■■■■□■■■■■□□■■■■□□□□■□■■■■■■■■■	700775747413771	702	EAI6-BGD1	Dar es Salaam	F/44	Not recorded
TZA0220111N2080	2011	N2080	■■■■■■■■■■■■□■■■■■■■■■■■□□□□□□■□□□□■■■□■■■	777737774020731	727	H1	Ngorongoro	F/41	negative
TZA0220121B0719	2012	B0719	■■■■■■■■■■■■■■■■■■■■□□□□■■□□□□■■□□□□■■■□■■■	777777606060731	815	LAM11-ZWE	Bunda	M/40	negative
TZA0220121N1238	2012	N1238	■■■■■■■■■■■■■■■■■■■■□□□□■■□□□□■■□□□□■■■□■■■	777777606060731	815	LAM11-ZWE	Ngorongoro	F/45	negative
TZA0220121B0729	2012	B0729	■■■■■■■■■■■■■■■■■■■■□□□□■■■■■□■■□□□□■■■■■■■	777777607660771	891	LAM9	Bunda	F/31	negative
TZA0220121B0735	2012	B0735	■■■■■■■■■■■■■■■■■■■■□□□□■■■■■□■■□□□□■■■■■■■	777777607660771	891	LAM9	Bunda	M/25	negative
TZA0220101N010-7	2010	N010-7	□□□■■■■■■■■■■■■■■■■■■■■■■■■■□□□□■□■■■■■■■■■	077777777413771	1090	EAI5	Ngorongoro	M/20	negative
TZA0220111B0604	2011	B0604	□□□■■■■■■■■■■■■■■■■■■■■■■■■■□□□□■□■■■■■■■■■	077777777413771	1090	EAI5	Bunda	M/36	negative
TZA0220101N010-9	2010	N010-9	■■■■■■■■■■■■■■■■■■■■□■■■■■■■■■■■□□■■■■■■■■■	777777677763771	1192	Manu2	Ngorongoro	F/35	negative
TZA0220111B0304	2011	B0304	■□□■■■■■■■■■■■■■■■■■■■■■■■■■□□□□■□■■■■□■■■■	477777777413671	1369	EAI5	Bunda	F/37	negative
TZA0220101N1062	2010	N1062	■■■■■■■□■■■■■■■■■■■■□□□□■■□□□□■■□□□□■■■□■■■	775777606060731	1549	LAM11-ZWE	Ngorongoro	F/39	negative
TZA0220111B0573	2011	B0573	■■■■■■■□■■■■■■■■■■■■□□□□■■□□□□■■□□□□■■■□■■■	775777606060731	1549	LAM11-ZWE	Bunda	F/32	positive
TZA0220121D0152	2012	D0152	■■■□□□□■■□■■□■■■■■■□□□□□□□□□□□□□□□□■■■■■■■■	703337400001771	1765	CAS1-Kili	Dar es Salaam	M/41	Not recorded
TZA0220121N1125	2012	N1125	■■■■■■■■■■■■■■■■■■■■■■■■■■■■□□□□■□■■■■■□■■□	777777777413730	1801	EAI1-SOM	Ngorongoro	M/22	positive
TZA0220101S002M	2010	S002M	■■■■■□□□□□■■■■■■■■■■■■■■■■■■■■■■□□□□■■■□□□■	760377777760701	1824	T1	Serengeti	M/54	negative

TZA0220101S0025	2010	S0025	■■■■■□□□□□■■■■■■■■■■■■■■■■■■■■■■□□□□■■■□□□■	760377777760701	1824	T1	Serengeti	M/41	negative
TZA0220111D0129	2011	D0129	■□□■■■■■■■■■■■■■■■■■■■■■■■■□□□□□■□■■■■■■■■	477777777013771	1957	EAI5	Dar es Salaam	F/33	Not recorded
TZA0220111B0657	2011	B0657	■■■■■■■■■■■■■■■■□■■■□□□□■■□□□□■■□□□□■■■□■■■	777775606060731	2196	LAM11-ZWE	Bunda	F/42	negative
TZA0220121B0659	2012	B0659	■■■■■■■■■■■■■■■■□■■■□□□□■■□□□□■■□□□□■■■□■■■	777775606060731	2196	LAM11-ZWE	Bunda	F/30	negative
TZA0220121S0711	2012	S0711	■■■■■■■■■■■■■■■■□■■■□□□□■■□□□□■■□□□□■■■□■■■	777775606060731	2196	LAM11-ZWE	Serengeti	F/43	negative
TZA0220111S0702	2011	S0702	■■■■■■■■■■■■■■■■□■■■□□□□■■□□□□■■□□□□■■■□■■■	777775606060731	2196	LAM11-ZWE	Serengeti	M/30	negative
TZA0220111S0706	2011	S0706	■■■■■■■■■■■■■■■■□■■■□□□□■■□□□□■■□□□□■■■□■■■	777775606060731	2196	LAM11-ZWE	Serengeti	M/42	negative
TZA0220111S0708	2011	S0708	■■■■■■■■■■■■■■■■□■■■□□□□■■□□□□■■□□□□■■■□■■■	777775606060731	2196	LAM11-ZWE	Serengeti	M/31	positive
TZA0220121N1247	2012	N1247	■■■□□□□□■□■■■■■■■■■■□□□□□□□□□□□□□□□■■■■■■■	701377400001771	2267	CAS	Ngorongoro	M/23	negative
TZA0220101N1171	2010	N1171	■■■□□□□■■□■■■■■■■■■■□□□□□□□□□□□□□□□□■■■■■■■	703377400000771	2269	CAS	Ngorongoro	F/45	negative
TZA0220111N1071	2011	N1071	■■■□□□□■■■■□□□□□■■■■□□□□□□□□□□□□■■■■■■■■■	703601740003771	2364	CAS1-Delhi	Ngorongoro	F/22	negative
TZA0220111D0121	2011	D0121	■■■□□□□■■□■■■■■■■■■■□□□□□□□□□□□□□□■■■■■■■■■	703377400003771	2391	CAS1-Kili	Dar es Salaam	M/29	Not recorded
TZA0220121N1011	2012	N1011	■■■□■■■■■□■■■■■■■■■□■■■■■■■■■■■■□□□□■■■■■■■	737377677760771	2484	T1	Ngorongoro	F/22	positive
TZA0220121N1132	2012	N1132	■■■■■■■■■■■■■■■■■■■■■■■■■■■■■■■■□□□□■■■■□□■	777777777760741	2736	T1	Ngorongoro	M/39	negative
TZA0220101B0306	2010	B0306	■□□□■■■■■■■■■■■■□□■■■■■■■■■■■■■■□□□□■■■□■■□	437774777760730	3880	T2-uganda	Bunda	M/38	positive
TZA0220111B0302	2011	B0302	■□□□■■■■■■■■■■■■□□■■■■■■■■■■■■■■□□□□■■■□■■□	437774777760730	3880	T2-uganda	Bunda	F/37	negative
TZA0220121B0720	2012	B0720	■□□■■■■■■■■■■■■■■■■■■■■■■■■■□□□□■□■■■□□□□■■	477777777413411	4056*	EAI5	Bunda	F/22	negative
TZA0220121B0722	2012	B0722	■□□■■■■■■■■■■■■■■■■■■■■■■■■■□□□□■□■■■□□□□■■	477777777413411	4056*	EAI5	Bunda	M/38	negative
TZA0220121N1253	2012	N1253	■■■■■■■■■□□□□□■■■■■■■■■■■■■■■■■■□□□□■■■■■■■	777017777760771	4057*	T1	Ngorongoro	M/55	negative
TZA0220121B0398	2012	B0398	■□□■■■■■■■■■■■■■■■■■■■■■■■■■□□□□■□■■□■■■■■■	477777777413371	4058*	EAI5	Bunda	M/41	negative
TZA0220101N1240	2010	N1240	■□□■■■■■■■■■■■■■■■■■■■■■■□□□□□□□□■■■■■■■■	477777776003771	Or01	EAI	Ngorongoro	M/37	negative
TZA0220101B0046	2010	B0046	■■■□■■□■■□■■■□■■■■■■■■■■■■■■■■■■□■■■■■■■■■	733357777773771	Or02	Manu1	Bunda	M/32	negative
TZA0220111N1251	2011	N1251	■■■■■■■■■□■■■□■■■□■■□□□□■■■■■■■■□□□□■■□■■■■	777356607760671	Or03	LAM9	Ngorongoro	F/40	negative
TZA0220121N1278	2012	N1278	■■■□■■□■■■■□■□□■■■■■□■■■□■□■□□□□□□□■■■■■■■■	733647672403771	Or04	EAI	Ngorongoro	M/30	negative
TZA0220111N1246	2011	N1246	■□■□□■■■□□■■■□■■■■■■■■■■■■■■■□□□□■□■■■■■□■■■	516357777413731	Or05	EAI1-SOM	Ngorongoro	F/21	negative
TZA0220101N1244	2010	N1244	■■■■■■□■■□■□■□■■■■■■■■□■■■■■■■■■■□□□■■■■■■■	773257677743771	Or06	Manu2	Ngorongoro	M/39	negative
TZA0220121N1370	2012	N1370	■■■■■■□■■□■□■■■■■■■■□□□□■■□■■■■■□□■■■■□■■■■	773277606763671	Or07	Manu2	Ngorongoro	F/44	negative
TZA0220121N1371	2012	N1371	■■■■■■■■■□■■■□■■■■■■■□■■■■■□□■□■■□□■■■■■■■■	777357676263771	Or08	Manu2	Ngorongoro	M/29	negative
TZA0220111N1160	2011	N1160	■■■■■■□■■□■■■□■■■■■■■■■■■■■□□□□□□□□■■■■■■■■	773357776003771	Or09	EAI	Ngorongoro	M/22	negative
TZA0220121N1266	2012	N1266	■■■■■■■■■■■■■■■■■■■■■■■■■■■■■□□□□□□■■■■■■■	777777777601771	Or10	Manu2	Ngorongoro	F/35	negative
TZA0220101N1057	2010	N1057	■■■■■■■■■■■■■■■■■■■■□□■■■■■■■□■■■□□■■■■■■■■	777777637563771	Or11	Manu2	Ngorongoro	M/24	negative
TZA0220111N1155	2011	N1155	■■■■■■□□□□■□□□■■■■■■■■■■■■■■□■□□■□■■■■■■■■□□	770217777513760	Or12	Manu1	Ngorongoro	M/27	negative
TZA0220111N1135	2011	N1135	■■■□□□□□□□□□□□□□■□□■□□□■□□□□■■□■□□□□□□■■□□□□	700002210320300	Or13	H3	Ngorongoro	F/37	positive
TZA0220101N1129	2010	N1129	■■■□□□□■■□■□■□□■□■□□□□■□□□□□□□□□□□□□■■□■□□□	703245020000640	Or14	CAS	Ngorongoro	F/49	negative
TZA0220111N1134	2011	N1134	■■■■■■■■■■■■■□□■■■■■■■■■■■■■■■■■□□■■■■■■■■■	777747777763771	Or15	Manu2	Ngorongoro	F/53	negative
TZA0220101B0660	2010	B0660	■□□□■■■■■■■■■■■■■□■■■■■■■■■■■■■■□□□□■■■□■■□	437775777760730	Or16	T2-uganda	Bunda	M/51	negative
TZA0220111B0713	2011	B0713	■■□□■■■■■■■■□■■■□□■□□■■■■■■■■■■■□□□□■■■□■■□	637734477760730	Or17	T2-uganda	Bunda	F/42	negative
TZA0220101B0393	2010	B0393	□□□■■■■■■■■■■■■■■■■■■■■■■■■■□□□□■□■■□■■■■■■	077777777413371	Or18	EAI5	Bunda	M/35	negative
TZA0220121B0723	2012	B0723	■■□□■■■■■■■■□■■■□■■□□■■■■■■■■■■■□□□□■■■□■■□	637735477760730	Or19	T2-uganda	Bunda	F/29	positive
TZA0220101B0542	2010	B0542	■□□■■■■■■■■■■■■■■■□■□■■■■■■■□□□□■□■■■■■□■■■	477777277413731	Or20	EAI8-MDG	Bunda	M/23	negative
TZA0220111B0590	2011	B0590	■□■■■■■■■■■■■■■■■■■■■■■■■□□□□□■■□□□□■■■■■■■	577777774060771	Or21	T1	Bunda	M/31	negative
TZA0220101B0389	2010	B0389	■■□□■■■■■■■■■■■■■■■□□■■■■■■■■■■■□□□□■■■□■■□	637777477760730	Or22	T2-uganda	Bunda	F/25	negative
TZA0220111D0132	2011	D0132	■■■□□□□■■■■■■■■■□■□□□□□□□□□□□□□□□□□■■■■■■■■	703775000003771	Or23	CAS	Dar es Salaam	M/45	Not recorded
TZA0220111D0119	2011	D0119	■■■■■■■■■■■□■■■■■■■■■■■□■■■■■□□□□■□■■□■■■■■■■	777677757413371	Or24	EAI6-BGD1	Dar es Salaam	F/21	Not recorded
TZA0220121D0163	2012	D0163	■■■□□□□■■□■■□□□□□■□□□□□□□□□□□□□□□□■■■■■■■	703301000001771	Or25	CAS	Dar es Salaam	F/61	Not recorded
